# The multistep oxidation of cholesterol to pregnenolone by human cytochrome P450 11A1 is highly processive

**DOI:** 10.1016/j.jbc.2023.105495

**Published:** 2023-11-24

**Authors:** Kevin D. McCarty, Lu Liu, Yasuhiro Tateishi, Hannah L. Wapshott-Stehli, F. Peter Guengerich

**Affiliations:** Department of Biochemistry, Vanderbilt University School of Medicine, Nashville, Tennessee, USA

**Keywords:** cytochrome P450, enzyme kinetics, enzyme mechanisms, steroid metabolism, cholesterol, electron transfer, oxidation-reduction, kinetic isotope effects

## Abstract

Cytochrome P450 (P450, CYP) 11A1 is the classical cholesterol side chain cleavage enzyme (P450_scc_) that removes six carbons of the side chain, the first and rate-limiting step in the synthesis of all mammalian steroids. The reaction is a 3-step, 6-electron oxidation that proceeds *via* formation of 22*R*-hydroxy (OH) and 20*R*,22*R*-(OH)_2_ cholesterol, yielding pregnenolone. We expressed human P450 11A1 in bacteria, purified the enzyme in the absence of nonionic detergents, and assayed pregnenolone formation by HPLC-mass spectrometry of the dansyl hydrazone. The reaction was inhibited by the nonionic detergent Tween 20, and several lipids did not enhance enzymatic activity. The 22*R*-OH and 20*R*,22*R*-(OH)_2_ cholesterol intermediates were bound to P450 11A1 relatively tightly, as judged by steady-state optical titrations and *k*_off_ rates. The electron donor adrenodoxin had little effect on binding; the substrate cholesterol showed a ∼5-fold stimulatory effect on the binding of adrenodoxin to P450 11A1. Presteady-state single-turnover kinetic analysis was consistent with a highly processive reaction with rates of intermediate oxidation steps far exceeding dissociation rates for products and substrates. The presteady-state kinetic analysis revealed a second di-OH cholesterol product, separable by HPLC, in addition to 20*R*,22*R*-(OH)_2_ cholesterol, which we characterized as a rotamer that was also converted to pregnenolone at a similar rate. The first oxidation step (at C-22) is the slowest, limiting the overall rate of cleavage. *d*_3_-Cholesterol showed no kinetic deuterium isotope effect on C-22, indicating that C-H bond cleavage is not rate-limiting in the first hydroxylation step.

The synthesis of all steroids proceeds from the cleavage of the 6-carbon tail from cholesterol by cytochrome P450 (P450, CYP) 11A1, the “side-chain cleavage” enzyme (P450_scc_) (Fig. 1). This mitochondrial enzyme, localized in adrenals and other hormone-producing organs, is highly regulated, and genetic variations can cause congenital adrenal insufficiency (1). Even though this enzyme has a well-defined function in steroid biosynthesis, it also catalyzes reactions with other substrates, including 7-dehydrocholesterol, other steroids (2, 3), vitamin D_3_ (4), and even some drugs (5).

**Figure 1:**
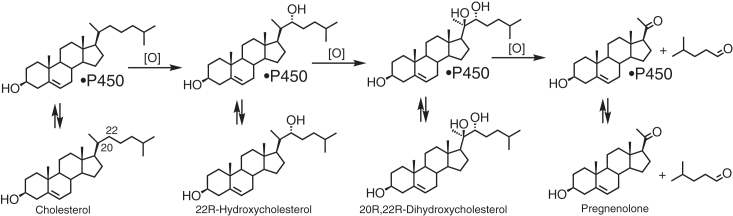
**The three-step oxidation of cholesterol to pregnenolone catalyzed by P450 11A1.** The equilibria between free and enzyme-bound steroids are shown and are the subject of this investigation.

Successful catalysis of P450 11A1 reactions requires reducing equivalents delivered by the mitochondrial P450 redox partners, adrenodoxin (Adx) and adrenodoxin reductase (AdR). Electrons (derived from NADPH) are shuttled through the flavin domains of the membrane-bound AdR protein to the soluble iron-sulfur matrix protein Adx. Electrons are subsequently delivered to P450 11A1 by Adx, thus facilitating the activation of molecular oxygen by P450.

X-ray crystal structures of P450 11A1 have been published (6, 7). All three steps of the reaction (Fig. 1) are considered to involve the high valent oxygen species known as compound I (FeO^3+^), based on cryoreduction/annealing data which demonstrated the catalytic competence of artificially formed Compound I (8, 9). Further details of the unusual oxidative cleavage of the 20*R*,22*R*-(OH)_2_ sterol by P450 11A1—that is, nucleophilic attack of a C-20 or C-22 hydroxyl group on Compound I—been provided through ^18^O-labeling studies (10), although an alternate explanation of the results has also been presented, based on theoretical calculations (11).

P450 11A1 has been studied for many years, with much of the early work involving the enzyme isolated from bovine adrenals. The isolation procedures vary, and many include the use of nonionic detergents, with the recombinant human enzyme as well as bovine P450 11A1. The assays that have been used for measuring enzyme activity (formation of pregnenolone) also vary considerably, including radioimmunoassay (12, 13), radio-TLC (14), gas chromatography (of derivatives) (15), and radio-HPLC (16). Not surprisingly, then, the reported activities of the purified bovine and human enzymes are quite variable in these assays, ranging from 0.29 to 34 nmol pregnenolone formed min^−1^ (nmol P450)^−1^ (12, 16, 17, 18, 19, 20, 21).

An important question, which we now address, is how processive the overall 3-step reaction is, that is, how likely each product is to dissociate from the enzyme before acting as a substrate for the next oxidation step. Early work on the enzyme has been reviewed by Lieberman and Lin (22). No hydroxy (OH) intermediates (between cholesterol and pregnenolone) were found in some cases (14, 16) but were seen in other steady-state studies, at low levels (15, 23), or in a reaction in which the enzyme was artificially cycled (24). Sugano *et al.* (21) reported that the intermediate 20*R*,22*R*-(OH)_2_ cholesterol could be detected as an intermediate when 22*R*-OH cholesterol was incubated with P450 11A1 but only when relatively low concentrations of Adx (*i.e.*, equimolar) were used. The authors interpreted the result as indicating that the binding of 20*R*,22*R*-(OH)_2_ cholesterol was weaker when lower concentrations of this accessory protein Adx were used. Tuckey and Cameron (25) were also able to convert 22*R*-OH cholesterol to 20*R*,22*R*-(OH)_2_ in human placental mitochondria. Lambeth *et al.* (12) concluded that each oxidation step occurs at approximately the same rate at 37 °C.

Although the synthetic steroid intermediates in the reaction pathway (Fig. 1) have been available for several years, the binding of all of these to P450 11A1 has not been investigated rigorously, in our opinion. Orme-Johnson *et al.* (26) reported *K*_d_ values of 4.9 and 81 nM for the bovine P450 11A1 complexes with 22*R*-OH cholesterol and 20*R*,22*R*-(OH)_2_ cholesterol, values obtained using equilibrium dialysis. Jefcoate reported a *K*_d_ of 0.40 to 0.45 μM for 20*R*,22*R*-(OH)_2_ cholesterol with the rat enzyme (27). Lambeth *et al.* (12) also considered the binding of the hydroxycholesterols, expressing *K*_d_ values in the context of a sterol to phosphatidylcholine ratio. Adx was reported to strongly enhance binding. The authors indicated that the rates of both binding and dissociation of 20*R*,22*R*-(OH)_2_ cholesterol with bovine P450 11A1 were >300 s^−1^ (based on attempts to use stopped-flow kinetics). They also concluded, based on the spectral sterol/phospholipid ratio data, that the “…intermediates are bound 2 to 3 orders of magnitude more tightly than cholesterol,” although even with a typical *k*_on_ rate of 10^6^ M^−1^ s^−1^ (28, 29) and a *k*_off_ rate of 300 s^−1^ would only correspond to a *K*_d_ of 300 μM (and *k*_on_ rate of 10^7^ M^−1^ s^−1^ to a *K*_d_ value of 30 μM).

The availability of recombinant human P450 11A1, its accessory proteins Adx and AdR and the intermediates in the reaction pathway (Fig. 1; Figs. S1–S8) allowed for the systematic investigation of the kinetic processivity of P450 11A1, using features of a general approach that we have applied with other multistep P450s (30, 31, 32), most recently with human P450 51A1 (33). We have also used a detergent-free preparation of the recombinant human enzyme to address questions about the contributions of lipids (and detergents) and the role of the accessory protein Adx in binding ligands, as well as C–H bonding breaking as a rate-limiting step. A kinetic model for the reaction was developed based on the results. We also report the formation and cleavage of a second conformer of 20*R*,22*R*-(OH)_2_ cholesterol in the normal reaction, as revealed by rapid-quench kinetics and HPLC separation.

## Results

### Expression, purification, and assays

We had expressed and purified human P450 11A1 previously, with the use of the nonionic detergent pentaethylene glycol monooctyl ether (C8E5) (3). We experienced low expression yields for P450 11A1 and used a different vector (pET23C+) for this study, using the same amino acid sequence as before (Fig. S9). The new vector notably employs a T7 promoter (Fig. S10) which recruits the T7 RNA polymerase for faster and more efficient transcription. Typical yields of P450 11A1 were 150 nmol purified enzyme (liter culture)^−1^.

Nonionic detergents are inherently difficult to remove from P450 enzymes (34). Accordingly, we had concerns about the presence of any residual nonionic detergent in the preparation, which might interfere in a careful study of the interactions of the enzyme with substrates. Although most of the P450 11A1 literature protocols utilize nonionic detergents, (mainly Tween 20) in purification (and even in assays), our preliminary assays showed that Tween 20 was inhibitory. However, we also noted that attempts to remove Tween 20 from preparations (*e.g.*, polystyrene beads, adsorption to hydroxylapatite and other chromatography columns followed by washing and elution) resulted in (i) enzyme precipitation or (ii) incomplete removal of the Tween 20 (as assayed colorimetrically (35)). Even under the best conditions, P450 11A1 preparations still contained ∼2 mg Tween 20 (nmol P450)^−1^.

We developed a procedure (suggested by Prof. Donghak Kim and Changmin Kim, Konkuk University, Seoul) in which the cell membranes were lysed in the presence of the ionic detergent CHAPS and applied to an Ni^2+^-nitrilo triacetate affinity column. After washing to remove CHAPS, the protein (electrophoretically homogeneous, Figs. S11 and S12) could be eluted with a high imidazole concentration and remained soluble.

**Figure 2:**
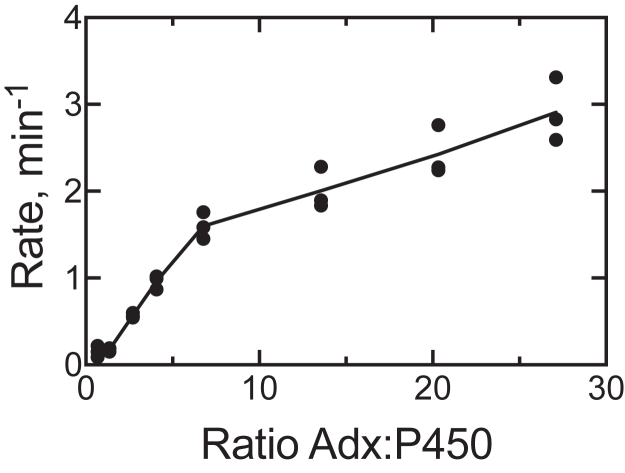
**Dependence of catalytic activity on Adx concentration.** Incubations (5 min, 37 °C) were run with 0.25 μM P450 11A1 (0.5 μM AdR), 50 μM cholesterol, and a variable concentration (0.17–6.8 μM) of Adx and were initiated with an NADPH generating system (Experimental procedures). Reactions were run in triplicate and the conversion of cholesterol to pregnenolone was calculated and plotted (nmol pregnenolone formed min^−1^ (nmol P450)^−1^). AdR, adrenodoxin reductase; Adx, adrenodoxin; P450, cytochrome P450.

**Figure 3:**
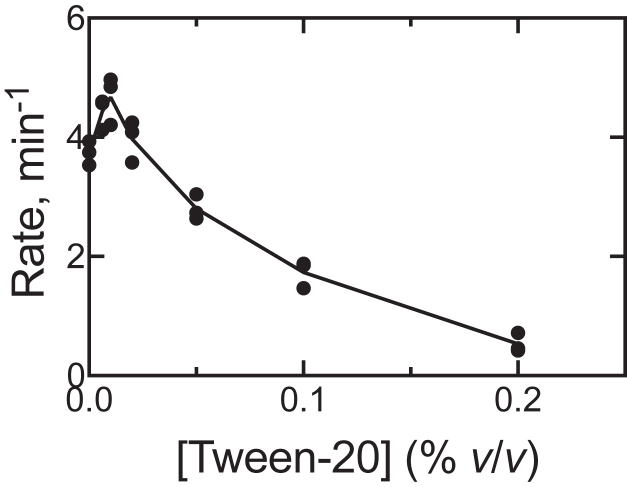
**Effect of Tween 20 on P450 11A1 catalytic activity.** Incubations (5 min, 37 °C) were run with 0.25 μM P450 11A1 (0.5 μM AdR) and 50 μM cholesterol with an excess (40-fold) of Adx and were initiated with an NADPH generating system (Experimental procedures). Reactions were run in triplicate and the conversion of cholesterol to pregnenolone was calculated and plotted (nmol pregnenolone formed min^−1^ (nmol P450)^−1^). (Note: These assays were done with P450 11A1 purified in the absence of Tween 20.). AdR, adrenodoxin reductase; Adx, adrenodoxin; P450, cytochrome P450.

**Figure 4:**
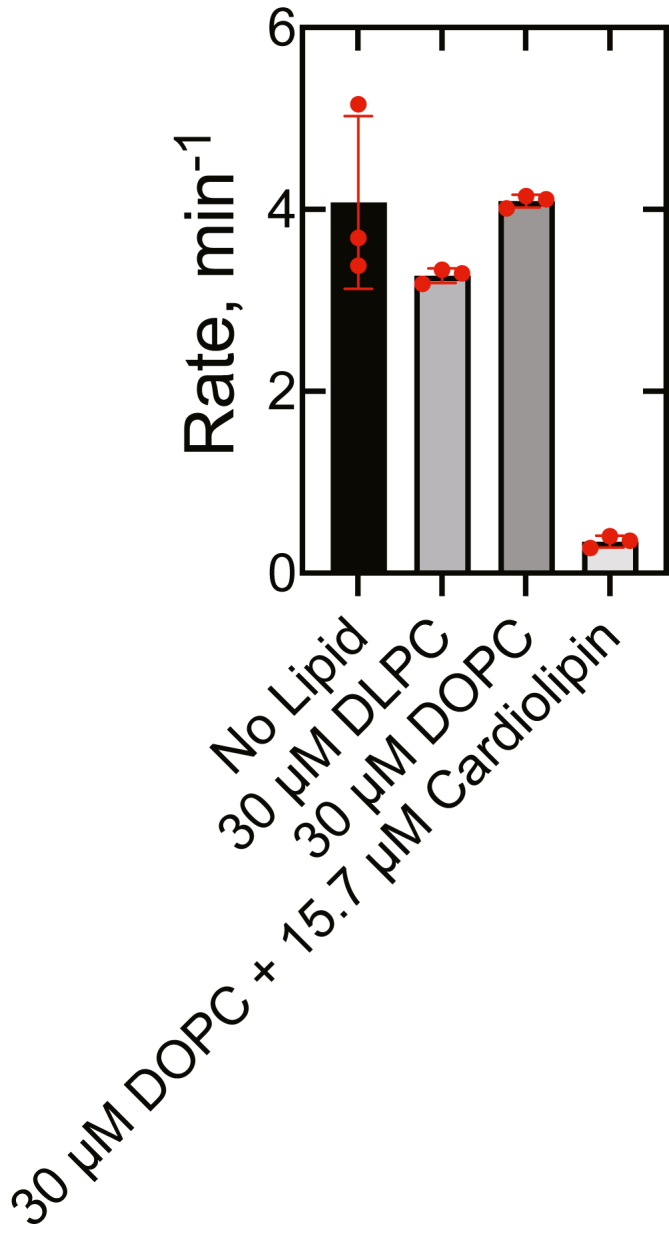
**Effects of lipids on P450 11A1 activity.** Incubations (5 min, 37 °C) were run with 30 μM phospholipid, 0.25 μM P450 11A1 (0.5 μM AdR), and 50 μM cholesterol with an excess (40-fold) of Adx and were initiated with an NADPH generating system (Experimental procedures). Reactions were run in triplicate and the conversion of cholesterol to pregnenolone was calculated and plotted (nmol pregnenolone formed min^−1^ (nmol P450)^−1^). AdR, adrenodoxin reductase; Adx, adrenodoxin; P450, cytochrome P450.

The substrate cholesterol and its 22*R*-OH and 20*R*,22*R*-(OH)_2_ products were delivered in 2-hydroxypropyl-β-cyclodextrin (HPCD) (36) in assays, circumventing the need to use any detergents or organic solvents. P450 11A1 readily coupled with the electron carrier Adx, a soluble protein, and the concentration of Adx was optimized (Fig. 2) (37). Tween 20 was found to be inhibitory at all concentrations >0.008% (w/v), under these conditions (Fig. 3). When P450 11A1 was solubilized with CHAPS and purified, the rate of the conversion of cholesterol to pregnenolone was routinely ∼5 to 8 min^−1^ (*i.e.*, nmol pregnenolone formed min^−1^ (nmol P450)^−1^), which is as high or higher than many (but not all (20)) previous values. Further, the activity was not enhanced by the addition of L-α-dilauroyl-*sn*-glycero-3-phosphocholine or L-α-dioleyl-*sn*-glycero-3-phosphocholine and was inhibited by a combination of L-α-dioleyl-*sn*-glycero-3-phosphocholine and cardiolipin (Fig. 4), in contrast to literature on the bovine enzyme (12, 38, 39, 40).

Accordingly, all steady-state assays were done, unless otherwise noted, with a P450 11A1:Adx:AdR molar ratio of 1:40:2 (Fig. 2, that is, 0.25 μM P450, 10 μM Adx, and 0.5 μM AdR), in the absence of added lipid or detergent, and with the addition of sterol substrates in HPCD (stock 45% w/v).

As mentioned in the Introduction, many types of assays have been used to monitor the steady-state conversion of cholesterol to pregnenolone (12, 14, 15, 16, 17, 18, 19, 20, 21). A reliable and sensitive method was required that could be used for presteady-state reactions and was not reliant on the presence of isotope in cholesterol, in that, we also worked with 22*R*-OH cholesterol and 20*R*,22*R*-(OH)_2_ cholesterol as substrates. We utilized a method based on our work with P450 17A1 (41, 42), in which we derivatized the product pregnenolone as its dansyl hydrazone and separated it by ultra-performance liquid chromatography (UPLC) with detection by positive ion electrospray mass spectrometry (MS). The steroid dehydroepiandosterone (DHEA) was used an internal standard.

### Steady-state kinetics

**Figure 5:**
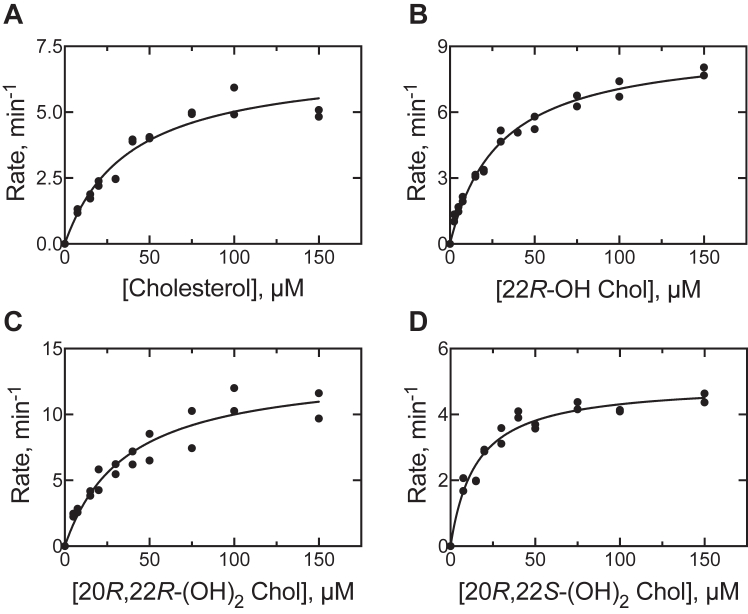
**Steady-state kinetics of oxidation of cholesterol, 22*R*-hydroxycholesterol, 20*R*,22*R*-dihydroxycholesterol, and 20*R*,22*S*-dihydroxycholesterol to pregnenolone by P450 11A1.** The individual substrates were incubated with the reconstituted P450 11A1 system (0.25 μM P450, 0.5 μM AdR, and 10 μM Adx), initiated with an NADPH generating system (Experimental procedures), and the product pregnenolone was extracted, derivatized, and quantified by UPLC-MS. Chol: cholesterol. *A*, cholesterol: *k*_cat_ 6.9 ± 0.5 min^−1^, *K*_m_ 38 ± 6 μM, *k*_cat_/*K*_m_ 0.18 ± 0.03 μM^−1^ min^−1^. *B*, 22*R*-OH cholesterol: *k*_cat_ 9.1 ± 0.3 min^−1^, *K*_m_ 29 ± 3 μM, *k*_cat_/*K*_m_ 0.31 ± 0.03 μM^−1^ min^−1^. *C*, 20*R*,22*R*-(OH)_2_ cholesterol: *k*_cat_ 14 ± 1 min^−1^, *K*_m_ 36 ± 7 μM, *k*_cat_/*K*_m_ 0.38 ± 0.07 μM^−1^ min^−1^. *D*, 20*R*,22*S*-(OH)_2_ cholesterol: *k*_cat_ 5.0 ± 0.2 min^−1^, *K*_m_ 15 ± 2 μM, *k*_cat_/*K*_m_ 0.33 ± 0.05 μM^−1^ min^−1^. See Table 1. Rates are presented on the *y*-axis as nmol product (pregnenolone) formed min^−1^ (nmol P450)^−1^. Incubations were run in duplicate and the conversion of sterol to pregnenolone was calculated and plotted. The nonlinear regression fits include the Prism error estimates for internal fitting (SE). The estimated parameters were not corrected using a quadratic equation, due to high *K*_m_ values relative to enzyme concentration. AdR, adrenodoxin reductase; P450, cytochrome P450; UPLC, ultra-performance liquid chromatography.

The dansyl derivatization method provided a sensitive means by which one can monitor the formation of pregnenolone in steady-state assays (Fig. 5). The kinetic analyses showed that the oxidations of cholesterol and its intermediates (22*R*-OH and 20*R*,22*R*-(OH)_2_ cholesterol) were similar in both *k*_cat_ and *k*_cat_/*K*_m_, with the 20*R*,22*R*-(OH)_2_ sterol being ∼2-fold greater in both parameters relative to the starting substrate. Interestingly, while 20*R*,22*S*-(OH)_2_ cholesterol—a nonnative substrate—was nearly 3-fold lower in *k*_cat_ than the native diol, it was roughly equal in *k*_cat_/*K*_m_ indicating that it is oxidized almost as efficiently as the native substrate (Table 1). P450 11A1 also oxidized [1,2-^3^H] cholesterol at a similar rate to that which we report using the dansyl derivatization method (Fig. S13), giving us further confidence in the accuracy of our LC-MS based approach.

**Table 1:** Oxidation and binding kinetic parameters of cholesterol and intermediates with P450 11A1

Steroid	*K*_d_, μM		*k*_cat_, s^−1^	*K*_m_, μM	*k*_cat_/*K*_m_, M^−1^ s^−1^	*k*_off_, s^−1^
	(−) Adx	(+) Adx				
Cholesterol	0.12 ± 0.01	0.11 ± 0.01	0.12 ± 0.01	38 ± 6	3.2 ± 0.5 × 10^3^	0.24 ± 0.01
22*R*-OH Chol	0.02 ± 0.01	0.020 ± 0.003	0.15 ± 0.01	29 ± 3	5.2 ± 0.5 × 10^3^	0.044 ± 0.001
20*R*,22*R*-(OH)_2_ Chol	0.03 ± 0.02	0.04 ± 0.01	0.23 ± 0.02	36 ± 7	6.4 ± 1.2 × 10^3^	0.39 ± 0.01
20*R*,22*S*-(OH)_2_ Chol	0.12 ± 0.03	0.09 ± 0.02	0.083 ± 0.003	15 ± 2	5.5 ± 0.8 × 10^3^	0.40 ± 0.01
Pregnenolone	0.48 ± 0.22	0.56 ± 0.12	N/A	N/A	N/A	0.55 ± 0.01

All kinetic parameters were calculated from experimental measurements. *k*_cat_ is the rate of conversion of the corresponding sterol to pregnenolone by P450 11A1. Enzymatic efficiency is reported as *k*_cat_/*K*_m_ (specificity constant).

Abbreviation: Chol, cholesterol.

### Steady-state binding of steroids to P450 11A1

**Figure 6:**
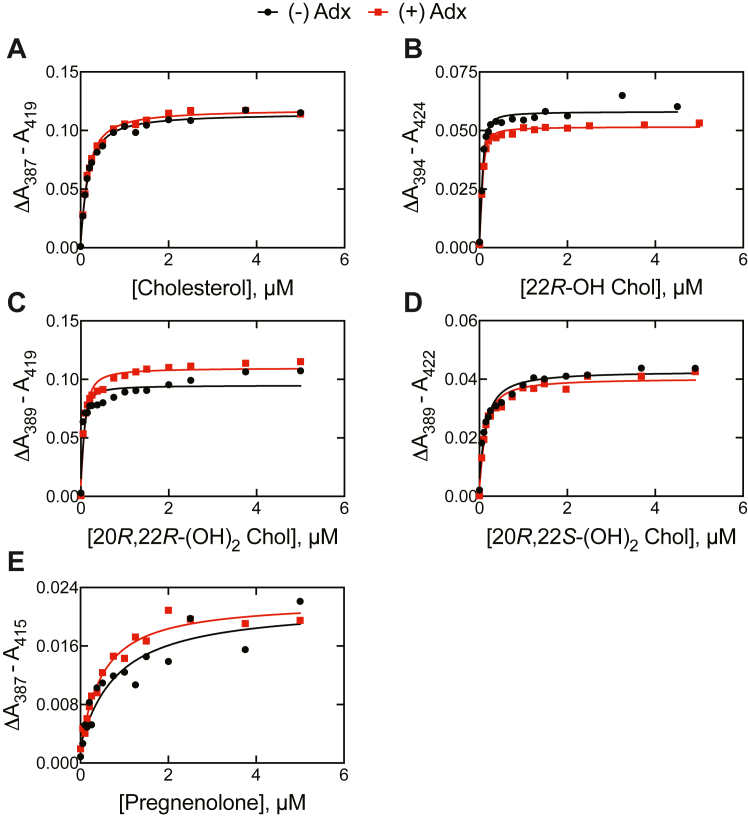
**Spectrally-determined *K***_**d**_**values of P450 11A1-steroid complexes.** Measurements were made with 0.10 μM P450 11A1 in a 10 cm cuvette both minus (•, *black*) or in the presence of (0.10 μM) Adx (▪, *red*). The titration curves are shown in the insets. Chol: cholesterol. *A*, cholesterol: *K*_d_ 0.12 ± 0.01 μM without Adx, 0.11 ± 0.01 μM with Adx. *B*, 22*R*-OH cholesterol: *K*_d_ 0.02 ± 0.01 μM without Adx, 0.020 ± 0.003 μM with Adx. *C*, 20*R*,22*R*-(OH)_2_ cholesterol: *K*_d_ 0.03 ± 0.02 μM without Adx, 0.04 ± 0.01 μM with Adx. *D*, 20*R*,22*S*-(OH)_2_ cholesterol: *K*_d_ 0.12 ± 0.03 μM without Adx, 0.09 ± 0.02 μM with Adx; *E*, pregnenolone: *K*_d_ 0.48 ± 0.22 μM without Adx, 0.56 ± 0.12 μM with Adx. See Table 1. Each data point corresponds to one measurement in singlet. Adx, adrenodoxin; P450, cytochrome P450.

Ligand binding constants were determined from the low-to high-spin (hypsochromic) spectral transition that occurs at the P450 heme as substrate entering the active site replaces water as the sixth axial heme ligand. This so-called “Type I” shift (43) was observed in the binding of each of the studied sterols to P450 11A1 (Fig. 6). Binding titrations were performed in a 10-cm cell with low concentrations (0.1 μM) of P450 due to our estimation of low *K*_d_ values in preliminary experiments. The *K*_d_ values calculated for all sterols were sub-μM (when quadratic fitting was applied) but were notably below the enzyme concentration for the oxidation intermediates (22*R*-OH and 20*R*,22*R*-(OH)_2_), indicating that these measurements likely contain error. As a result, we subsequently measured *k*_off_ values of the P450-sterol complexes, which were consistent with the low *K*_d_ values observed (see below). Binding titrations were repeated in the presence of Adx (1 M equivalent, Fig. 6), although no significant effect was observed, even when Adx was added in excess (10-fold with cholesterol as the substrate, data not shown). The enzymatic byproduct of cholesterol side chain cleavage (4-methylpentanal) did not induce a spectral shift with P450 11A1, even at comparably high (up to 1 mM) concentrations.

### Binding of Adx to P450 11A1 ± cholesterol

**Figure 7:**
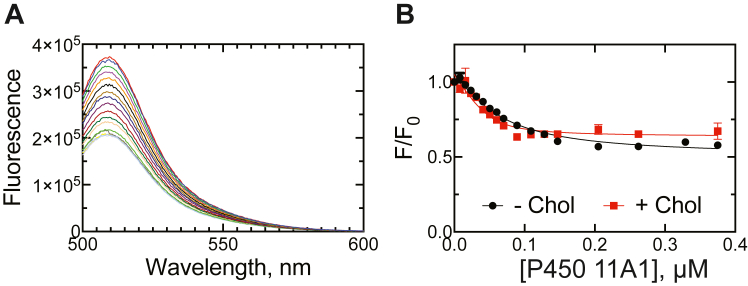
**Effect of cholesterol on P450 11A1 binding to Adx.***A*, titration of P450 11A1 (0–400 nM) into Alexa 488-Adx (50 nM) gradually quenched the fluorophore and yielded a decrease in fluorescence output (between 500 and 600 nm). Spectra were collected after each addition of P450. *B*, normalized fluorescence (F/F_0_) of titrations of P450 11A1 with (▪, *red*) and without (•, *black*) cholesterol (equimolar). A 1:1 M complex of P450 and cholesterol was used as titrant, where indicated. Data were fit to a quadratic equation. Adx, adrenodoxin; P450, cytochrome P450.

Because Adx has been reported to stimulate the binding of P450 11A1 to its substrates (12, 44), we examined whether the binding of detergent-free P450 11A1 to Adx is enhanced by cholesterol, utilizing our fluorescently-labeled Adx construct (Alexa 488-Adx) (45). A 1:1 M complex of P450 11A1 and cholesterol was gradually titrated into a solution containing Alexa 488-Adx (50 nM), and the quenching of Adx fluorescence was monitored over the course of the reaction. We found that cholesterol stimulated (∼4.5-fold) the binding of P450 11A1 to Adx, as observed by a moderate reduction in the dissociation constant (*K*_d_) from 38 [CI 29–49] nM (without sterol) to 8.2 [CI 3.7–16] nM (with sterol) (Fig 7). We note that these values are similar to the *K*_d_ values of 24 nM (without sterol) and 8.2 nM (with sterol) reported by Yablokov *et al.* (46) for human P450 11A1 and the value of 13.4 nM (without sterol) reported by Schiffler *et al.* (47) for bovine P450 11A1, both using surface plasmon resonance methods.

### Steroid *k*_off_ rate measurements

**Figure 8:**
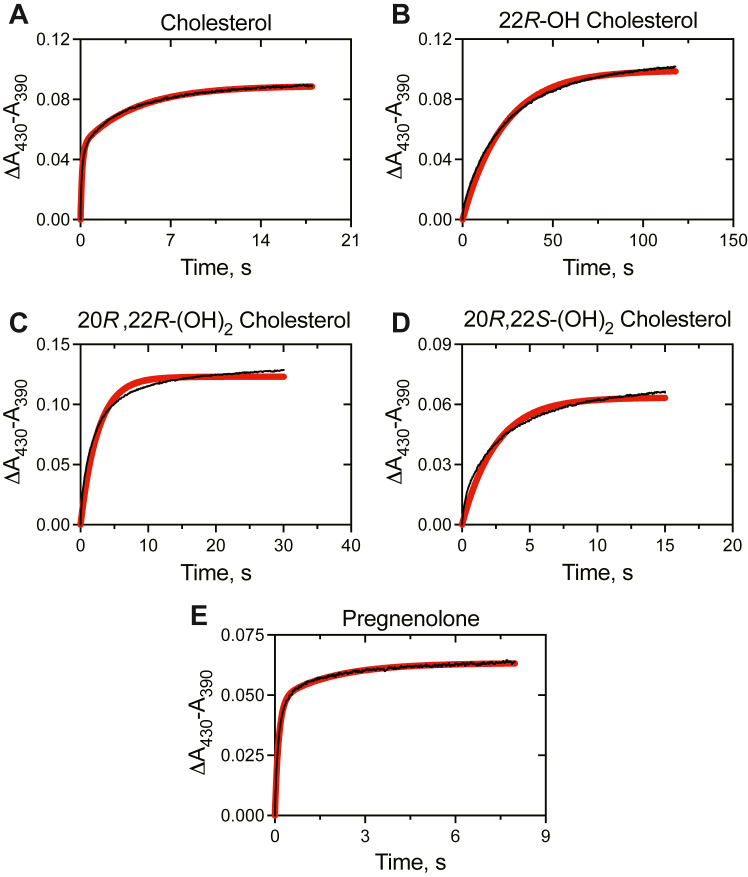
***k***_**off**_**measurements of P450 11A1-steroid complexes.** In each case, an equimolar concentration of P450 11A1 (2 μM) and each steroid (2 μM), in one syringe, was mixed with a 20 μM concentration of ketoconazole in the other syringe, in an OLIS RSM-1000 stopped-flow spectrophotometer. Full spectra were collected and the *A*_390_ and *A*_430_ data were used in the calculations. *A*, cholesterol: 0.24 ± 0.01 s^−1^; (*B*), 22*R*-OH cholesterol: 0.044 ± 0.001 s^−1^; (*C*), 20*R*,22*R*-(OH)_2_ cholesterol: 0.39 ± 0.01 s^−1^; (*D*), 20*R*,22*S*-(OH)_2_ cholesterol: 0.40 ± 0.01 s^−1^; (*E*), pregnenolone: 0.55 ± 0.01 s^−1^. Fits were made to single (*B*–*D*) or double (*A* and *E*) exponentials using the GraphPad Prism program, following transfer of x, y data files, and the error estimates are also from that program. The *red lines* show the data traces and the thinner *black lines* are the fits with the indicated rates. See Table 1. A minimum of eight replicate traces were averaged to yield each plot. P450, cytochrome P450.

Ketoconazole is a broad spectrum P450 inhibitor that is known to bind tightly to P450 11A1 (48). Interaction of the azole nitrogen atom with the P450 heme produces a bathochromic (“type II”) spectral shift (to ∼430 nm)—an inverse effect of the binding of substrate. The rapid binding of ketoconazole to unliganded P450 11A1 (≥5 × 10^5^ M^−1^ s^−1^, data not shown) and the low *K*_d_ values observed for the studied sterols afforded the opportunity to use the inhibitor in a “trap” experiment, wherein solutions of aqueous ketoconazole (20 μM) and P450-sterol complex (1:1, 2 μM each) are mixed on a rapid timescale (diluting all reaction components 2-fold). Upon mixing, free enzyme (yielded from the dissociation of the P450-sterol complex) is rapidly captured by aqueous ketoconazole, and the resulting P450-inhibitor complex is detected by the corresponding type II spectral transition. The rate of ketoconazole binding to the free enzyme (*k*_on_) is then an estimate for the rate of dissociation of the P450-sterol complex, which we report as *k*_off_ (Fig. 8).

The *k*_off_ rate was measured for each sterol in the reaction pathway (Fig. 1). Plots for the cholesterol intermediates (22*R*-OH and 20*R*,22*R*-(OH)_2_) were monophasic with *k*_off_ rates of 0.04 and 0.39 s^−1^, respectively (Table 1). Higher *K*_d_ values for cholesterol and pregnenolone (Fig. 6) yielded biphasic plots in the trap experiment, owing to a greater fraction of unliganded enzyme available at equilibrium. Biexponential fitting of the cholesterol and pregnenolone data yielded fast phases that were consistent with the ketoconazole *k*_on_ rate (7.1 and 7.9 × 10^5^ M^−1^ s^−1^, respectively) and slow phases corresponding to dissociation of the P450-sterol complex (*k*_off_) prior to ketoconazole trapping (0.24 and 0.55 s^−1^, respectively). For all the sterols we examined except 22*R*-OH cholesterol, the experimentally determined steady-state forward rate of oxidation was slightly slower than the *k*_off_ rate. The *k*_off_ rates were not measured in the presence of Adx as we did not observe an effect of the redox partner when determining ligand dissociation constants (*K*_d_, see above).

Measurement of *k*_on_ rate(s) was not performed because the sterols are delivered in a solution of HPCD, from which the dissociation rate is not known. However, for the purpose of method validation, cholesterol (20 μM in HPCD) was mixed with P450 11A1 and an apparent *k*_on_ rate of 2.5 × 10^3^ M^−1^ s^−1^ was observed (*i.e.*, 0.05 s^−1^ at 20 μM final concentration), which is >200-fold slower than the *k*_on_ rate of ketoconazole to unliganded P450 (in the presence of HPCD).

### Single turnover kinetics

The reaction of P450 11A1 with cholesterol was followed in a “single-turnover” experiment to measure the rates of formation and disappearance of the oxidized intermediates in the reaction pathway (Fig. 1). As the accumulation of reaction intermediates is characteristic of a distributive reaction, the analysis of intermediate lifetimes is a powerful approach for gauging reaction processivity. In this experiment, enzyme and substrate are preincubated in a 1:1 stoichiometric ratio before reaction initiation—hence “single-turnover.” We have employed the use of rapid quench kinetics to perform this analysis with P450s 2E1, 19A1, 17A1, 11B2, and—most recently—51A1 (30, 31, 32, 33, 49).

**Figure 9:**
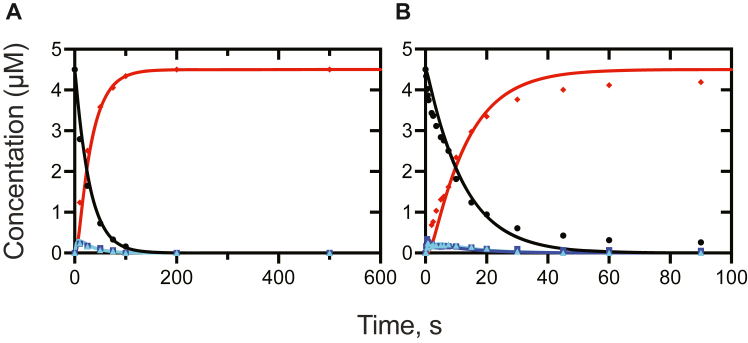
**Single-turnover time course of the reaction of a complex of P450 11A1 and [1,2-**^**3**^**H] cholesterol.***A*, the reaction was initiated by mixing of NADPH (5 mM) from one syringe with a complex of 5 μM P450 11A1, 5 μM Adx, 10 μM AdR, and 4.5 μM [1,2-^3^H]-cholesterol (HPCD complex) in the other. At each of the indicated times, the reaction was quenched with 1 M HCl and the reaction products were extracted and quantified using radio-HPLC as described in the Experimental procedures. Cholesterol (•—•, *black line*); 22*R*-OH cholesterol (▪—▪, *dark blue line*); 20*R*,22*R*-(OH)_2_ cholesterol (▲—▲, *light blue line*); pregnenolone (♦—♦, *red line*). Lines are fit to the kinetic mechanism in Table 2 and the indicated rate constants. *B*, as in Part *A*, with lines fit to the kinetic mechanism in Table 2 and the indicated rate constants, but with the concentration of Adx in the experiment raised to 50 μM. Each data point is the aggregate of five pooled reactions analyzed as a single sample. AdR, adrenodoxin reductase; Adx, adrenodoxin; HPCD, 2-hydroxypropyl-β-cyclodextrin; P450, cytochrome P450.

In this reaction, a 5 μM complex of P450 11A1 and 4.5 μM cholesterol was assembled, and Adx was added at either equimolar (low, 5 μM) or 10-fold excess (high, 50 μM) concentrations (along with 10 μM AdR). The complex was diluted 2-fold (with 5 mM NADPH) in the apparatus to initiate the reaction, which was quenched at variable times. The products of the reaction were extracted and quantified by radio-HPLC. At low Adx concentrations, the reaction was complete in 3 min with a half-life (*t*_1/2_) of ∼17 s for the disappearance of cholesterol (Fig. 9*A*). The reaction was stimulated by excess Adx and was complete in 1 min with a cholesterol *t*_1/2_ of ∼8 s (Fig. 9*B*). Reducing the Adx concentration to be equimolar with P450 (*i.e.*, 5 μM) slightly increased (∼2-fold) the concentrations of intermediates that accumulated during the reaction (Fig. 9). Overall, however, both reactions rapidly proceeded to final product and no substantial accumulation of intermediate products was observed. (The analysis of kinetic modeling is described below.)

**Figure 10:**
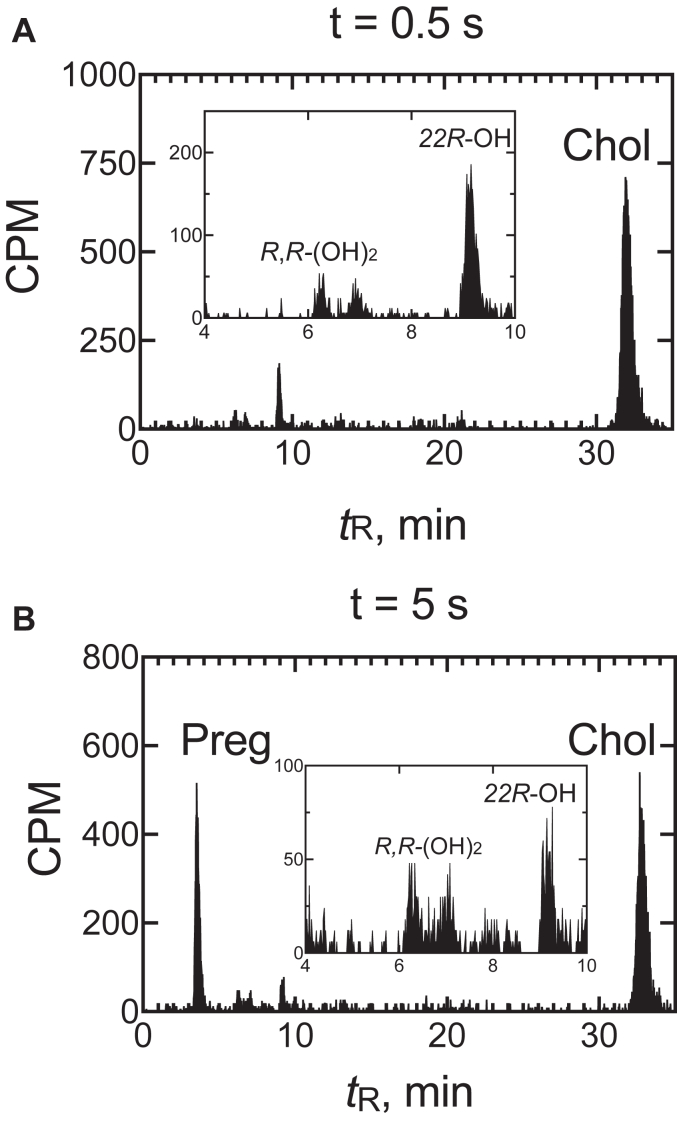
**Radio-HPLC of reaction of P450 11A1 with [1,2-**^**3**^**H]-cholesterol under single-turnover conditions and separation of a second dihydroxycholesterol intermediate.** Radio-HPLC data from the reaction in Part *B* of Figure 9. *A*, chromatogram from HPLC analysis of sample at t = 0.5 s; *B*, chromatogram from HPLC analysis of sample at t = 5 s. The insets show the separation of 22*R*-OH cholesterol (*t*_R_ 9.3 min) and the two conformers of 20*R*,22*R*-(OH)_2_ cholesterol (*t*_R_ 6.2 and 6.8 min). P450, cytochrome P450.

**Figure 11:**
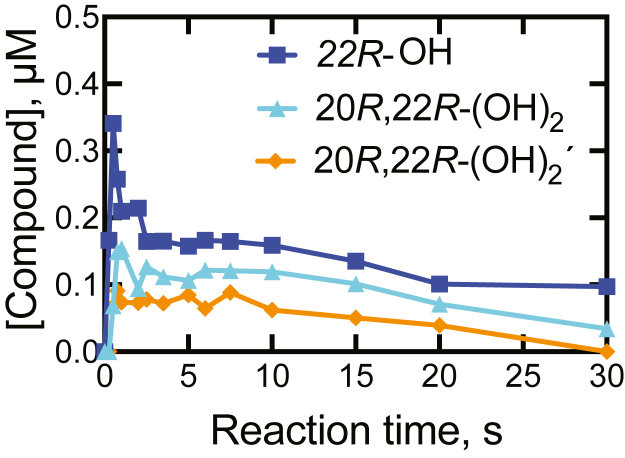
**Expansion of the single turnover data from the reaction in**Figure 10**.***A*, 22*R*-OH cholesterol (▪—▪, *dark blue line*); 20*R*,22*R*-(OH)_2_ cholesterol (*t*_R_ 6.2 min, Figure 10, ▲—▲, *light blue line*), and the alternate 20*R*,22*R*-(OH)_2_ cholesterol conformer (20*R*,22*R*-(OH)_2_ cholesterol', *t*_R_ 6.8 min, Figure 10, ♦—♦, *tan line*). Each data point is the aggregate of five pooled reactions analyzed as a single sample.

**Figure 12:**
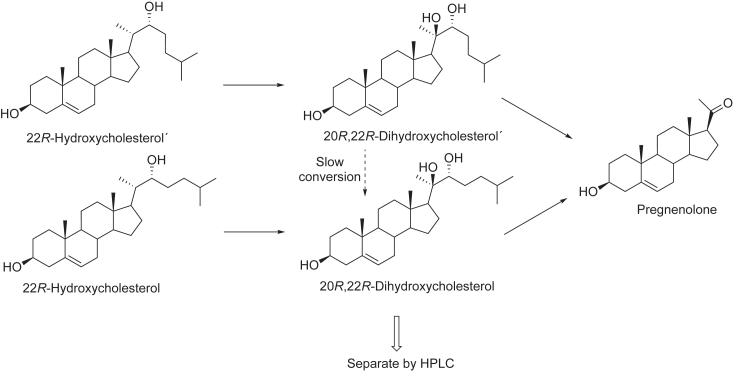
**Proposed explanation for 20*R*-hydroxylation of 22*R*-OH cholesterol to form rotamers of 20*R*,22*R*-(OH)**_**2**_**cholesterol.** 22*R*-OH cholesterol' and 20*R*,22*R*-(OH)_2_ cholesterol' represent less stable conformers of 22*R*-OH and 20*R*,22*R*-(OH)_2_ cholesterol, respectively.

The products of cholesterol oxidation were cleanly resolved with our HPLC separation method, where the order of elution was pregnenolone/20*R*,22*R*-(OH)_2_ cholesterol/22*R*-OH cholesterol/cholesterol (Fig. 10). In the single-turnover study with ^3^H-cholesterol, two peaks were reproducibly seen in the HPLC zone corresponding to 20*R*,22*R*-(OH)_2_ cholesterol, at all reaction time points between 0.5 to 5 s (Fig. 10). Both of these radioactive peaks showed similar kinetics of formation and disappearance (Fig. 11). One of these migrated with standard 20*R*,22*R*-(OH)_2_ cholesterol, but the possibility was considered that the later eluting peak might be 20*S*,22*R*-(OH)_2_ cholesterol (neither eluted with synthetic 20*R*,22*S*-(OH)_2_ cholesterol). A mixture of the two ^3^H-labeled peaks was subjected to treatment with NaIO_4_ (which cleaves *vic*-diols), and both peaks disappeared, having converted to pregnenolone (Fig. S14). However, in control experiments without added NaIO_4_, the peak with the later *t*_R_ was converted to the earlier *t*_R_ peak, that is, 20*R*,22*R*-(OH)_2_ cholesterol. This experiment was repeated four times with similar results. We conclude that the two peaks are not diastereomers but are conformers (rotamers) with a *t*_1/2_ for conversion on the order of hours for conversion to the more stable form (of 20*R*,22*R*-(OH)_2_ cholesterol) under our conditions (Fig. 12).

### Kinetic isotope effect assays

The results of the single turnover kinetics suggested that the first step of cholesterol oxidation by P450 11A1 is slow and potentially rate-limiting. We synthesized [20, 22, 22]-*d*_3_ cholesterol (∼32% *d*_3_) to investigate whether C-H bond breaking in the first hydroxylation was rate-limiting. P450 11A1 incubations were performed under (low Adx) single-turnover conditions (5 μM P450, 5 μM cholesterol; see Experimental procedures) with *d*_0_- (authentic, unlabeled) and *d*_3_-cholesterol, and the disappearance of each was monitored by LC-atmospheric pressure chemical ionization-MS (*m/z* 369.3516 and 372.3705, respectively). In theory, if C-H bond breaking were rate-limiting in the initial hydroxylation then the rate of *d*_3_-cholesterol consumption would be slower relative to the *d*_0_ compound due to the increased strength of a C-D bond. Although the *d* substitution of the cholesterol at C-22 was not complete, the observation of the loss of the *d*_3_ isotopologue of cholesterol provides a measurement of the first reaction step under single turnover conditions, in that multiple reaction cycles are not involved.

**Figure 13:**
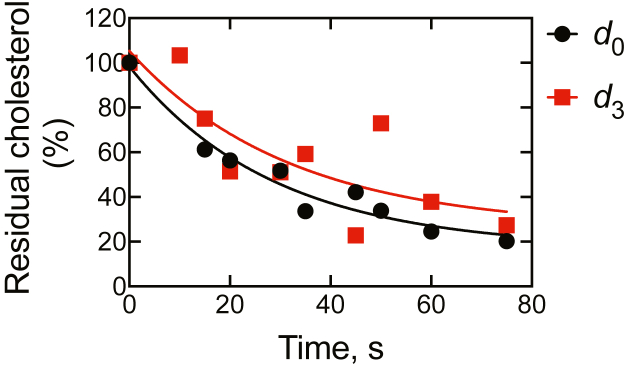
**Lack of a kinetic isotope effect in cholesterol 22*R*-hydroxylation.** The disappearance of *d*_0_-cholesterol (•—•, *black line*, 5 μM) and *d*_3_-cholesterol (♦—♦, *red line*, 5 μM) is plotted against reaction time. The disappearance of substrate is normalized to the 0 s control reactions with P450 11A1 (5 μM). Both plots were fit to nonlinear (exponential decay) regression models for comparison. The rates observed in this experiment with *d*_0_-and *d*_3_-cholesterol were 0.035 and 0.032 s^−1^, respectively. Incubations were run in singlet and rates were calculated and plotted. P450, cytochrome P450.

We did not observe a kinetic isotope effect. Both *d*_0_-and *d*_3_-cholesterol were oxidized at roughly the same rate, as judged by sterol half-lives (*t*_1/2_) of 20 s and 22 s, respectively (Fig. 13). A significant (*k*_H_/*k*_D_ ≥ 2) isotope effect would be obvious under our conditions (especially at the earlier time points), based on kinetic simulations in KinTek Explorer (https://kintekcorp.com/software). We conclude that C-H bond breaking is not rate-limiting in the 22*R*-hydroxylation of cholesterol by P450 11A1.

### Kinetic modeling

**Figure 14:**
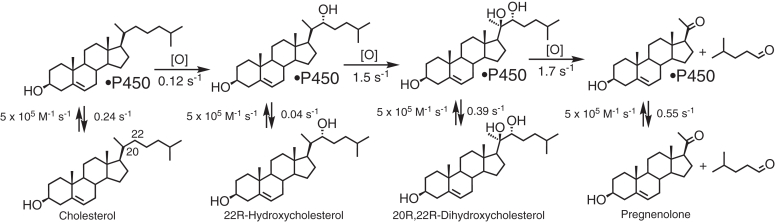
**Three-step oxidation of cholesterol to pregnenolone by P450 11A1 and estimated rate constants for individual steps.** The same rate constant was used for binding of all ligands to P450 11A1 (5 × 10^5^ M^−1^ s^−1^). See Table 2 and Figs. S15 and S16. P450, cytochrome P450.

A minimal kinetic model was assembled to fit the data of the single-turnover reactions (Fig. 9) using the parameters listed in Table 2 (Fig. 14). In the model (Figs. S15 and S16), the sterol *k*_on_ rates (*k*_1,_ *k*_3,_ *k*_5,_ *k*_7_) were set to the experimentally determined value for P450 11A1 and ketoconazole (5.3 × 10^5^ M^−1^ s^−1^) and sterol *k*_off_ rates (*k-*_1,_ *k-*_3,_ *k-*_5,_ *k-*_7_) were those measured by stopped-flow spectroscopy (Table 1 and Fig. 8). Each oxidation reaction is irreversible, and thus the rates of the reverse reactions (*k*_-2_, *k*_-4_, *k*_-6_) were set to zero. The single-turnover data (Fig. 9) were fit to a single exponential to calculate the first-order decay of cholesterol, that is, the rate of cholesterol 22*R*-hydroxylation (*k*_2_). This rate (0.09 s^−1^, when 10-fold (50 μM) excess Adx was used) was comparable to our experimentally determined steady-state *k*_cat_ of pregnenolone formation from cholesterol (0.12 s^−1^, Fig. 5*A* and Table 1). The rate of 20*R*-hydroxylation of 22*R*-OH cholesterol (*k*_4_) was estimated similarly to *k*_2_ from exponential fitting of first-order formation of the 20*R*,22*R*-(OH)_2_ sterol from the 22*R*-OH sterol. The rate of C-C bond cleavage (*k*_6_) was estimated as the steady-state rate determined earlier (Fig. 5*C* and Table 1). These parameters (*k*_2_, *k*_4_, and *k*_6_) were then adjusted by visual approximation to yield the values reported in Table 2 which were used for kinetic modeling as they gave the most satisfactory fits to the experimental data (Figs. 9, S15, and S16). Notably, the rate constants for both hydroxylation of 22*R*-OH cholesterol and C-C cleavage (Table 2) were much faster (1.5 s^−1^ and 1.7 s^−1^, respectively) than the steady-state *k*_cat_ values determined earlier (Fig. 5 and Table 1), attributed to the influence of other steps in the steady-state assays.

**Table 2:** Model and rate constants used in single turnover kinetic modeling

Reaction step	Steps	*k*^+^ (low, high Adx)	*k*^−^
E+S↔ES	*k*_1_, *k*_-1_	5.3 × 10^5^ M^−1^ s^−1^	0.24 s^−1^
ES→EP	*k*_*2*_, *k*_-2_	0.04, 0.12 s^−1^	0
E+P↔EP	*k*_*3*_, *k*_-3_	5.3 × 10^5^ M^−1^ s^−1^	0.044 s^−1^
EP→EQ	*k*_*4*_, *k*_-4_	0.4, 1.5 s^−1^	0
E+Q↔EQ	*k*_*5*_, *k*_-5_	5.3 × 10^5^ M^−1^ s^−1^	0.39 s^−1^
EQ→ER	*k*_*6*_, *k*_-6_	0.55, 1.7 s^−1^	0
E+R↔ER	*k*_*7*_, *k*_-7_	5.3 × 10^5^ M^−1^ s^−1^	0.55 s^−1^

E: P450 11A1, S: cholesterol; P: 22*R*-OH cholesterol; Q: 20*R*,22*R*-(OH)_2_ cholesterol; R: pregnenolone. *k*^+^: *k*_n_, *k*^-^: *k*_-n_.

## Discussion

A procedure was developed to purify human P450 11A1 in the absence of nonionic detergents, in order to avoid many of the previous complications with this enzyme. We used the purified human P450 11A1 to evaluate its properties and the kinetics—and thermodynamics—of several steps in the overall reaction and to reexamine some earlier reports in the literature. What emerges is a description of the 3-step side chain cleavage reaction in which a rate-limiting initial 22*R*-hydroxylation is followed by two rapid steps, and the *k*_off_ rate constants of all the intermediate products/substrates are low.

Much of the early work in the P450 11A1 field was done with the bovine enzyme, isolated from adrenal glands, so strict comparisons may not be possible. Our own measurements were made with recombinant bovine Adx, although our studies with other mitochondrial P450s have not shown any significant differences between bovine and human Adx (37). We were able to develop a method to solubilize recombinant human P450 11A1 with the ionic detergent CHAPS and then remove it by dialysis without the addition of Tween 20 or other nonionic detergents. The protein remained soluble and was catalytically active.

Tween 20 was strongly inhibitory to activity (Fig. 3). Our *k*_cat_ value for conversion of cholesterol to pregnenolone was 6.9 nmol product formed min^−1^ (nmol P450)^−1^ (at 40-fold excess Adx) (Table 1 and Fig. 5*A*). Rates in the literature vary at least from 0.29 (17) to an unusually high value of 34 (20) nmol product min^−1^ (nmol P450)^−1^ with the bovine and human enzymes, with almost all <10 min^−1^, utilizing a variety of other assay methods. Although much has been published about roles of specific lipids and phospholipids with P450 11A1 enzymes (12, 38, 39, 44), we observed no stimulation of activity by these under our conditions (Fig. 4). In fact, cardiolipin—a major phospholipid constituent of the mitochondrial inner membrane—was found to inhibit P450 11A1 activity. Although P450 11A1 is a membrane-bound enzyme, the electron carrier Adx is a soluble protein, and the assumption should not be made that P450 11A1 requires a lipid environment (at least when the substrates are delivered in cyclodextrins). This may be true in general for mitochondrial P450s; *e.g.*, with P450 27C1 we did not observe any effect of L-α-dilauroyl-*sn*-glycero-3-phosphocholine on initial rates (50). We have not compared *K*_m_ values in the literature, but these are only useful when coupled to *k*_cat_, that is, using the ratio *k*_cat_/*K*_m_ as a specificity constant (51).

Although reports have appeared that the presence of Adx enhances the affinity of P450 11A1 for its substrates by 10- to 20-fold (12, 44), we did not see this effect when we examined P450 11A1-Adx complexes (Fig. 6). (The *K*_d_ for a P450 11A1·Adx complex is low (Fig. 7), so most of these two proteins were complexed in these titrations (Fig. 6).) If the binding of cholesterol to P450 11A1 is not enhanced by Adx, then (using a “thermodynamic box” analysis (44, 52, 53)) the binding of cholesterol to P450 11A1 should not have a dramatic effect on the binding of Adx to P450 11A1, which was indeed the case in an assay using Adx tagged with a fluorescent dye, that is, a 4.5-fold change, which translates into <1 kcal mol^−1^ change in Gibbs free energy (ΔΔ*G* = −RT ln (*K*_d,1_/*K*_d,2_) (54) (Fig. 7). This observation is supported by Yablokov *et al.* (46), who found that the *K*_d_ of a (human) P450 11A1-Adx complex decreased from 24 nM to 8.2 nM (utilizing surface plasmon resonance) when cholesterol was added to the experiment, reporting very similar values to ours and constituting a roughly ∼3-fold decrease in *K*_d_ in the presence of cholesterol. Strushkevich *et al.* (7) similarly failed to observe a role of Adx in sterol binding to human P450 11A1, reporting no difference in binding affinities of P450 11A1-Adx fusion proteins for either cholesterol or the 22*R*-OH intermediate compared to the native enzyme (in the absence of Adx). These results can be contrasted with a study with the bovine enzyme, however, in which a 30-fold enhancement was reported using a different approach (44).

All of the hydroxy sterols, as well as cholesterol and pregnenolone, produced low-to high-spin changes that could be used to monitor spectral binding and to measure rates of release from P450 11A1. Lambeth *et al.* (53) reported that 22*R*-OH cholesterol caused a low-spin shift, but this conclusion is presumably due to the weaker spectral shift relative to other ligands (Fig. 6*B*). Previously reported *K*_*d*_ values for P450 11A1 ligands vary considerably (26, 27). We used a 10-cm spectrophotometer cell to lower the enzyme concentration for these assays, reducing the P450 11A1 concentration to 100 nM (Fig. 6), and applied quadratic fitting because of the low *K*_*d*_ values, which were as low as ∼20 nM (for 22*R*-OH cholesterol) (Fig. 6). Many of the values reported in the literature (with bovine P450 11A1) are considerably higher (27). The previous (bovine P450 11A1) values most similar to ours are actually those of Orme-Johnson *et al.* (26) (4.9 nM for 22*R*-OH cholesterol and 81 nM for 20*R*,22*R*-(OH)_2_ cholesterol), which is surprising in that these were obtained using equilibrium dialysis, a method not generally well-suited to highly insoluble ligands. It has been reported that the binding of the intermediate hydroxy sterols is 100- to 300-fold tighter than for cholesterol (12), but this difference was considerably less in our work (Table 1 and Fig. 6).

It was also reported (with the bovine enzyme (12)) that the association and dissociation of both cholesterol and 20*R*,22*R*-(OH)_2_ cholesterol were both complete within the dead time of a stopped-flow instrument, that is, *k* >300 s^−1^, but our dissociation rates are clearly much slower (Fig. 8). (because of the use of HPCD, we did not estimate binding (“on”) rates for the sterols, in that these likely reflect the dissociation from the cyclodextrin, at least in part.)

Some articles have reported the absence of hydroxy cholesterol intermediates between cholesterol and pregnenolone in P450 11A1 reactions (14, 16) but others have observed one or two (21, 23, 24). Sugano *et al.* (21) did only with a low concentration of Adx. Mast *et al.* (55) reported the presence of 22*R*-OH cholesterol in the bovine eye (retina and also pigment epithelium), which seems unusual in light of our work, but the possibility exists that another P450 (or a non-P450 oxidase) performs only this single hydroxylation. We detected intermediates in single turnover experiments, starting with cholesterol (Figs. 9 and 11). The levels of the intermediates were similar with 1:1 and 10:1 M ratios of Adx to P450 11A1 (Fig. 9). This result seems to contrast with that seen by Sugano *et al.* (21) for 20*R*,22*R*-(OH)_2_ cholesterol. The results could be fit to a model with high processivity, that is, a high ratio of the rate of each oxidation state to the product dissociation rate (Fig. 14). (The oxidation rate of C-C cleavage extracted from kinetic modeling (Fig. 14) is considerably faster than the value determined in the steady-state, which we attribute to a hereto unknown rate-limiting step.)

During analysis of the single turnover experiments, we detected a second radioactive peak (derived from cholesterol) with a retention time (*t*_R_) in the region of 20*R*,22*R*-(OH)_2_ cholesterol, that is, preceding both 22*R*- and 22*S*-OH cholesterol (Fig. 10). The amount of radioactivity in that peak was similar to that in the peak that migrated with standard 20*R*,22*R*-(OH)_2_ cholesterol. It did not migrate with standard 20*R*,22*S*-(OH)_2_ cholesterol. Radioactivity eluted with 22*R*-OH cholesterol, but not 22*S*-OH cholesterol (Fig. 10). It would be highly unlikely for the stereochemistry to change at the C-22 carbon in the course of the second hydroxylation (at C-20). The peak corresponding to the unknown was collected and treated with sodium periodate (NaIO_4_), after which all of the radioactivity migrated with pregnenolone. The result confirms that the unknown compound is a vicinal glycol, that is, a 20,22-(OH)_2_ cholesterol.

Based on the kinetic results with 20*R*,22*R*-(OH)_2_ cholesterol, we propose that P450 11A1 binds 22*R*-OH cholesterol in two forms (Fig. 12), which we do not really know the shapes of. Each form can be hydroxylated (*via* hydrogen abstraction at C-20) to give the corresponding conformer of 20*R*,22*R*-(OH)_2_ cholesterol. The two conformers of 20*R*,22*R*-(OH)_2_ cholesterol were formed at similar rates and were further oxidized (to cleave the side chain) at similar rates (Fig. 11). The two 20*R*,22*R*-(OH)_2_ cholesterol conformers are apparently stable to acid and to CH_2_Cl_2_ extraction (see Experimental procedures) and are separable by HPLC (Fig. 10), which is not unusual for rotamers (*e.g.*, formamidopyrimidine nucleic acids) (56, 57). Upon standing, however, one conformer (*t*_R_ 6.8 min, Fig. 11) converts to the more stable one (*t*_R_ 6.2 min in Fig. 10). To our knowledge, the formation of multiple, separable conformers has not been observed previously in P450 reactions.

The observation that multiple conformers of 20*R*,22*R*-(OH)_2_ cholesterol are intermediates in the reaction sequence complicates proposals about the mechanism of the C-C bond cleavage. We have proposed that this last step involves the reaction of compound I (FeO^3+^) with either the C-20 or C-22 OH (10) (^18^O label (from ^18^O_2_) was neither incorporated into pregnenolone nor the enzymatic byproduct, 4-methylpentanal), either of which may be possible, as is a theoretical proposal involving proton abstraction and selective transfer from the C-22 hydroxyl (11). A theoretical study (11) concluding a proton-coupled electron transfer mechanism is consistent with the ^18^O_2_-labeling results (10). However, the calculations in the theoretical paper (11) are based on crystal structures with either 22*R*-OH (6, 58) or 22*S*-OH cholesterol (58) or the synthetic 20*R*,22*R* isomer of (OH)_2_ cholesterol (58), which has generally been accepted to be the intermediate (15). However, the enzyme showed similar specificity constants for 20*R*,22*R*- and 20*R*,22*S*-(OH)_2_ cholesterol (Fig. 5). Most importantly, not only was 20*R*,22*R*-(OH)_2_ cholesterol identified as an intermediate in the single-turnover studies (Fig. 5) but what we identified as a conformer of the 20*R*,22*R*-isomer also was, and the rates of formation and cleavage were similar in the presteady-state work (Figs. 9 and 10). (Morisaki *et al.* (15) had also shown that other isomers—20*S*,22*S* and 20*S*,22*R*—could act as substrates of the enzyme, albeit being less efficient.) Accordingly, any mechanistic conclusions based on only the more stable 20*R*,22*R*-(OH)_2_ conformer are subject to caveats about interatomic distances, and so on.

In conclusion, our results indicate that P450 11A1 is not inherently dependent upon a lipid environment for catalytic activity and that the nonionic detergent Tween 20 inhibits the enzyme. The 3-step, 6-electron oxidation is a highly processive process due to the relatively low off-rates of the intermediates and the rapid oxidation of 22*R*-OH cholesterol and 20*R*,22*R*-(OH)_2_ cholesterol. We report an alternate and significant intermediate in the process, a conformer that arises from an alternate enzyme-substrate conformation in the second hydroxylation step of the reaction. The first hydroxylation (conversion of cholesterol to 22*R*-OH cholesterol) is rate-limiting in the overall sequence (Table 2 and Fig. 9). However, C–H bond breaking does not appear to be rate-limiting within that step (Fig. 13). The rate-limiting step within the reactions remains unknown; Schiffler *et al.* (47) reported that the rate of reduction of the bovine P450 11A1 by Adx is much faster than the oxidations (30–50 s^−1^), though the possibility that reduction of the human enzyme by its redox partner is slower cannot be excluded.

## Experimental procedures

### Chemicals

Cholesterol and pregnenolone were purchased from Millipore-Sigma-Aldrich and used without further purification. [1,2-^3^H]-Cholesterol was from American Radiolabeled Chemicals. 22*R*- and 22*S*-OH cholesterol were purchased from Steraloids. Dansylhydrazine was from Millipore-Sigma-Aldrich and used without further purification.

### Chemical synthesis

See Supporting information section for 20*R*,22*R*-(OH)_2_ cholesterol, 20*R*,22*S*-(OH)_2_ cholesterol, [20,22,22-^2^H_3_]-cholesterol (*d*_3_-cholesterol), and 4-methylpentanal.

### Preparation of steroid-HPCD solutions

Sterol solutions were prepared by adding dry powders directly to HPCD solution (45%, w/v). Mixtures were sonicated, heated (37 °C), and mixed with a vortex device until fully dissolved. When necessary, incubation was performed overnight with shaking (37 °C, 200 rpm) to ensure full incorporation into the cyclodextrin. Sterol solutions were stored at 4 °C and were heated and sonicated prior to use.

### Enzymes

Adx (59) and AdR (60) were expressed in *Escherichia coli* and purified as described previously. The expression and purification of P450 11A1 has been modified from previous procedures (3) and is described below.

#### Expression of P450 11A1

A P450 11A1 gene insert with a C-terminal His_6_ tag (codon-optimized) was digested and gel purified out of pCWori+ using the restriction enzymes NdeI and HindIII before being ligated into pET23C(+) (digested with the same enzymes) according to standard protocols. The P450 11A1 plasmid was transformed into *E. coli* C41(DE3) cells along with molecular chaperones GroES/GroEL according to standard heat shock procedures. An aliquot of the transformation was plated on LB agar supplemented with ampicillin and chloramphenicol and allowed to incubate (37 °C) overnight. A single colony was used to inoculate LB broth (100 ml) supplemented with ampicillin (100 μg ml^−1^) and chloramphenicol (20 μg ml^−1^). This preculture was incubated overnight with shaking (37 °C, 250 rpm). Bulk cultures (1 l, in 2.8-L Fernbach flasks) were prepared from terrific broth media supplemented with antibiotics (equivalent to the LB media) and arabinose (4 mg ml^−1^, for GroES/GroEL induction) and were inoculated with preculture (10 ml). Bulk cultures were grown (37 °C, 250 rpm, ∼3 h) until the OD_600_ reached ∼0.4 to 0.6, at which point P450 expression was induced by the addition of isopropyl β-D-1-thiogalactopyranoside (1 mM) and δ-aminolevulinic acid (1 mM) and flasks were supplemented with fresh ampicillin (50 μg/ml). Induced cultures were incubated (28 °C, 95 rpm, ∼48 h) until harvest by centrifugation (3500*g*, 30 min, 4 °C). Pellets were stored at −80 °C until use.

#### Purification of P450 11A1

Cell pellets were thawed on ice and gradually resuspended in a sonication buffer (100 ml per cell pellet (obtained from a one-liter culture)) composed of potassium phosphate buffer (100 mM, pH 7.4) containing glycerol (20%, v/v), Mg(C_2_H_3_O_2_)_2_ (6 mM), DTT (0.1 mM), and one cOmplete EDTA-free Protease Inhibitor Cocktail tablet (Roche) per 50 ml resuspension. Cells were lysed by sonication on ice in a 600 ml stainless steel beaker using a Model FB505 (500 W) probe instrument (Thermo Fisher Scientific) equipped with a bolt tip for 15 min (15 s on/45 s off, 45% intensity). The lysate was centrifuged (14,500*g*, 50 min, 4 °C) to remove debris and the supernatant was subjected to ultracentrifugation (10^4^*g*, 60 min, 4 °C) to collect membranes. Pellets were gently resuspended in a buffer composed of potassium phosphate buffer (100 mM, pH 7.4) containing glycerol (20%, v/v), EDTA (0.1 mM), 2-mercaptoethanol (10 mM), and sodium CHAPS (1%, w/v) and the resuspension was stirred (resolubilized) overnight (4 °C). The solution was again subjected to ultracentrifugation and the supernatant was applied to a metal affinity (Ni-nitrilo triacetate) column (1.5 cm × 8 cm, ∼15 ml) previously equilibrated with equilibration buffer (potassium phosphate buffer (100 mM, pH 7.4) containing glycerol (20%, v/v), NaCl (500 mM), and imidazole (20 mM)). The column was washed with equilibration buffer (10 column volumes), after which the concentration of imidazole in the buffer was increased to 50 mM (3 column volumes), 80 mM (3 column volumes), and finally 100 mM (3 column volumes) before elution of P450 with 150 mM imidazole (3 column volumes). The P450 band (red) began to migrate during the 100 mM wash and was fully displaced during the 150 mM elution. Column fractions were pooled based on absorbance (417 nm), concentrated with centrifugal concentrators (30 kDa molecular weight cut-off (to <10 ml), and dialyzed against a buffer of potassium phosphate buffer (200 mM, pH 7.4) containing glycerol (20%, v/v), and DTT (0.1 mM) using dialysis tubing (6–10 kDa molecular weight cut-off). P450 11A1 (<10 ml) was dialyzed against four volumes (1 l) of buffer, yielding a final maximal imidazole concentration <2 nM (Figs. S11 and S12). The material (20.3 μM) was aliquoted and stored at −80 °C until use.

### UV-visible spectroscopy

Spectra were recorded using either an OLIS-Cary 14 or OLIS-Aminco DW2a instrument (On-Line Instrument Systems) in the split-beam mode. The OLIS-Cary 14 instrument was used in the P450 11A1 titrations with a 10-cm cell (Starna Cells, catalog no. 34-Q-100, volume 25 ml).

### Binding of Alexa 488-Adx to P450 11A1

A previously described Alexa Fluor 488-labeled Adx construct and experimental protocol were used (45) to perform the fluorescence titration of P450 11A1 with Adx. Alexa 488-Adx (50 nM) was prepared in potassium phosphate buffer (100 mM, pH 7.4) in a 1 ml glass cuvette. P450 11A1 was gradually titrated (0–0.4 μM) into the cuvette (to a maximum of 2% (v/v)), and spectra were recorded using an OLIS DM-45 spectrofluorometer (1.24 mm slit width) with fluorophore excitation at 493 nm and scanning emission spectra from 500 to 600 nm. To investigate whether cholesterol affects the binding of Adx to P450 11A1, we performed the titrations with and without cholesterol. A 1:1 stoichiometric ratio of P450 11A1 and cholesterol (20 μM) was preformed and titrated into the cuvette. *K*_d_ values were calculated fitting plots of the normalized fluorescence at emission maximum *versus* concentration of P450 11A1 using a quadratic equation in GraphPad Prism.

### LC-MS assays of conversion of steroids to pregnenolone

Steady-state kinetic assays were performed with a P450:AdR:Adx molar ratio of 1:2:40, and preliminary experiments established optimal conditions for P450 11A1 activity. Reactions were performed by reconstituting P450 (0.25 μM), AdR (0.5 μM), and Adx (10 μM) on ice for 10 min prior to dilution in potassium Hepes buffer (50 mM, pH 7.4) and H_2_O and the addition of substrate (sterol, in HPCD (45% w/v)). Sterol dilutions were performed in HPCD to ensure that the HPCD composition was equal throughout all incubations (final 4.5% w/v). The reaction mixture was split between vials (425 μl in each), and reactions were preincubated (37 °C, 5 min) prior to initiation (75 μl) with an NADPH-generating system (0.5 mM NADP^+^, 10 mM glucose 6-phosphate, and 2 μg ml^−1^ glucose 6-phosphate dehydrogenase) (61). Reactions (5 min) were terminated by extraction into CH_2_Cl_2_ (2 ml) and transferred to an ice bath. After the experiment was complete, the quenched reactions were subjected to centrifugation (10^3^*g*, 5 min, 23 °C) to separate layers, the organic (bottom) layer of each sample was removed (1.6 ml) to fresh glass vials, and the samples were brought to dryness under a stream of nitrogen.

Steroid extracts were subsequently derivatized with dansylhydrazine and analyzed as described previously (42). Briefly, the dried residue was resuspended (200 μl) in C_2_H_5_OH (fortified with 0.1% v/v CF_3_CO_2_H, as the derivatization reaction is H^+^-catalyzed) and mixed with a vortex device. Samples were transferred to 1.5 ml amber glass vials, solutions of dansylhydrazine (10 mM) and DHEA, 10 μM, as internal standard) were added, and vials were capped, mixed with a vortex device, and incubated in the dark overnight (∼16 h, 23 °C). The derivatization reaction was quenched with acetone (50 mM), briefly incubated in the dark (∼30 min, 23 °C), and was then neutralized with base (250 μl of 0.5 M NaOH) and extracted into CH_2_Cl_2_ (1 ml). The samples were mixed with a vortex device and the organic (bottom) layer was removed (0.8 ml) to clean amber vials. Solvent was again removed under nitrogen, and the dried residue was dissolved in a mobile phase (100 μl) of 0.1% CF_3_CO_2_H in aqueous CH_3_CN (50%, v/v) for analysis.

Samples were injected (10 μl, held at 25 °C) on a 2.1 mm × 50 mm (1.7 μm) Acquity BEH octadecylsilane (C_18_) column (held at 40 °C) using a Waters Acquity UPLC with a flow rate of 0.2 ml min^−1^ and a gradient mobile phase of (A) 0.1% aqueous HCO_2_H and (B) CH_3_CN as follows (all %B, v/v): 0 min, 40%; 0.5 min, 40%; 8 min, 100%; 8.5 min, 95%; 9 min, 100%; 9.1 min, 40%; and 10 min, 40%. The column eluate was subjected to electrospray ionization (beginning at *t*_R_ 3.5 min to avoid the acetone dansyl hydrazone) and the product (pregnenolone dansyl hydrazone) was detected using a Waters QDa on-line mass spectrometer (Waters) set in the positive-ion electrospray mode using a cone voltage of 15 V, a sampling frequency of 10 Hz, and scanning from *m/z* 150 to 800. Data were processed using MassLynx software (Waters). Standard curves (of pregnenolone, with DHEA as internal standard) were prepared fresh for each assay, and product formation was assessed relative to the quotient of pregnenolone and DHEA peak areas.

### *K*_d_ determination

*K*_d_ values were determined by difference spectroscopy as previously reported (33, 43) but with some modifications. Briefly, a long-path quartz cuvette (10 cm, 25 ml) containing P450 11A1 (0.1 μM, in 100 mM potassium phosphate buffer (pH 7.4)) was prepared and a baseline was recorded in an OLIS-Cary 14 spectrophotometer. Where indicated, the redox partner Adx was added in equimolar concentration (0.1 μM) to P450. Sterol solutions (in 45% HPCD, w/v) were added to the cuvette, the contents of the cuvette were inverted to mix, and a spectrum was recorded (350–500 nm) after each addition. The total volume added to each cuvette was kept ≤2% (v/v, 0.25 ml) in the assay. The maximum absorbance difference (usually ΔA_390_−A_418_) was plotted against the total sterol concentration, and the data were fit to a quadratic equation in GraphPad Prism software (GraphPad, https://www.graphpad.com) to estimate the dissociation constant (*K*_*d*_). A quadratic equation was used to correct the ligand concentration for the enzyme-bound concentration (used as $Y=B+\frac{A}{2}*\frac{1}{E}*\left(\left(\text{Kd}+E+X\right)-\sqrt{{\left(\text{Kd}+E+X\right)}^{2}-\left(4*E*X\right)}\right)\;$ (or Y = B + (A/2)∗(1/E)∗((Kd + E + X)-sqrt((Kd + E + X)∧2 -(4∗E∗X)) in Prism software), where E = enzyme concentration, Kd = dissociation constant, and X = free sterol concentration).

### Single-turnover kinetics

#### Low Adx concentration (benchtop)

P450 11A1 (5 μM) was reconstituted with its redox partners AdR (10 μM) and Adx (5 μM) on ice (10 min) prior to dilution in potassium Hepes buffer (50 mM, pH 7.4) and H_2_O and the addition of ^3^H-cholesterol substrate (4.5 μM, 0.5 Ci/mmol). The concentration of substrate was deliberately kept slightly below the enzyme concentration to ensure that single-turnover conditions were strictly followed. The mixture (0.75 ml) was preincubated (37 °C, 5 min) in a shaking water bath prior to initiation with an equal volume of NADPH solution (5 mM, from a 10 mM stock in 50 mM potassium Hepes buffer, pH 7.4), thus reducing the concentrations of all enzymes and substrate to one-half their starting concentrations. After completing the desired incubation time, an aliquot (200 μl) was removed from the incubation and added to vials containing aqueous HCl (800 μl, 1 M). The quenched reactions were mixed with a vortex device and placed on ice. When the time course was complete, the quenched reactions were mixed with CH_2_Cl_2_ (5 ml) to extract reaction products and were centrifuged (10^3^*g*, 5 min, 23 °C) to separate layers, and the organic (bottom) layers were transferred (4 ml) to fresh glass vials. The extraction process was repeated and the two extracts (2 × 4 ml) from each sample were combined to maximize product recovery. Samples (8 ml) were brought to dryness under a stream of nitrogen, and the dried residues were dissolved in CH_3_OH (75 μl) and transferred to autosampler vials for analysis by radio-HPLC.

#### High Adx concentration (rapid quench)

Enzyme and NADPH solutions were prepared as described for the benchtop experiments (above) but with the modification that the concentration of Adx was increased to 50 μM (from 5 μM). Due to the stimulation of P450 11A1 cholesterol side-chain cleavage activity by high concentrations of Adx (Fig. 2), this reaction had to be monitored at sub-second intervals that were achievable with the use of a rapid quench-flow apparatus (KinTek). The rapid quench-flow is a rapid mixing device that combines equal volumes (∼19 μl) of two solutions (P450 and NADPH, held at 37 °C) into a central internal chamber to initiate a reaction. After the preset reaction time is completed, the mixture (∼38 μl) is then rapidly combined with a quench solution (1 M HCl, ∼160 μl), expelled from the apparatus, and collected in vials. Utilizing this experimental design, the reaction of enzyme-substrate complex (P450 11A1-Adx-AdR and ^3^H-cholesterol) was initiated with NADPH on a variable timescale (0–90 s), pooling five replicates of each time point to maximize sensitivity. The products of each quenched reaction (∼1 ml) were extracted with two volumes (5 ml) of CH_2_Cl_2_, brought to dryness, and dissolved in CH_3_OH as described for the benchtop experiments.

#### Radio-HPLC conditions

Samples (held at 4 °C) were injected (50 μl) using a Agilent 1100 Series HPLC system on a NovaPak (Waters) octadecylsilane (C_18_) 3.9 mm × 100 mm (4 μm) column (held at 25 °C) with a flow rate of 1.0 ml min^−1^ using a gradient mobile phase of (A) H_2_O and (B) CH_3_CN as follows (all in % B, v/v): 0 min, 70%; 10 min, 100%; 35.5 min, 100%; 37 min, 30%; and 40 min, 70%). The flow rate was increased to 1.5 ml min^−1^ (from the 10 min to 10.5 min interval) to decrease the retention of cholesterol and was later returned to 1.0 ml min^−1^ (from the 35 min to 35.5 min interval) to prepare for the next injection. The column eluate was mixed (1:2, v/v) with FlowLogic U scintillation cocktail (LabLogic) and radioactivity (^3^H) was detected using a β-RAM Model 5 radioflow detector (IN/US, LabLogic). Analytes were quantitated on the basis of their ^3^H peak areas relative to the summed radioactivity of the four compounds of interest.

### Kinetic isotope effect

The kinetic isotope experiment was designed similarly to the benchtop experiment described above, but with some modifications. P450 11A1 (5 μM) was reconstituted with the redox partners AdR (10 μM) and Adx (5 μM) on ice (10 min) prior to dilution in potassium Hepes (50 mM, pH 7.4) and H_2_O and addition of *d*_3_-cholesterol substrate (5 μM). The mixture was preincubated (5 min) prior to initiation with NADPH (2.5 mM, from 25 mM in potassium Hepes (50 mM, pH 7.4)) thus bringing all components to their desired final concentrations. After completing the desired incubation time, an aliquot (500 μl) was removed from the incubation and added to vials containing CH_2_Cl_2_ (5 ml) and the vials were mixed with a vortex device and placed on ice. The quenched reactions were centrifuged (10^3^*g*, 5 min, 23 °C) to separate layers, and the organic (bottom) layers were removed (4 ml) and transferred to fresh glass vials. The extraction process was repeated and the two extracts (2 × 4 ml) from each sample were combined to maximize product recovery. Samples (8 ml) were brought to dryness under a stream of nitrogen, and the dried residues were dissolved in CH_3_OH (25 μl) and transferred to autosampler vials for analysis.

Samples (held at 4 °C) were injected (10 μl) using a Waters Acquity UPLC system on a Acquity BEH octadecylsilane (C_18_) 2.1 mm × 50 mm (1.7 μm) UPLC column (held at 25 °C) with a flow rate of 0.5 ml min^−1^ using an isocratic mobile phase of CH_3_CN (only). The column eluate was subjected to atmospheric pressure chemical ionization and analyzed using a Thermo Fisher Scientific LTQ XL Orbitrap mass spectrometer instrument operating in positive mode, scanning from *m/z* 100 to 500, with a resolution setting of 120,000. Data were processed using Xcalibur QualBrowser (Thermo Fisher Scientific) software (version 2.0.7; https://www.thermofishcer.com/order/catalog/product/OPTON).

### *k*_off_ determination

Determinations of *k*_off_ rates were performed as previously described (33). Briefly, an OLIS-RSM 1000 stopped-flow spectrophotometer (held at 23 °C) with slit widths of 1.24 mm (8 nm bandpass) and scanning from 350 to 500 nm was loaded with solutions of (A) ketoconazole (20 uM, in 100 mM potassium phosphate buffer (pH 7.4)) and (B) P450 11A1 (2 μM, same buffer). These solutions were mixed (in equal volumes, giving a two-fold dilution of all reaction components), and the observed rate of ketoconazole binding (ΔA_430_) to P450 was 5.3 s^−1^. Then, sterol (in HPCD) was added in equimolar concentration (2 μM) to P450 11A1 and the mixture was loaded into the drive syringe and mixed with solution A (ketoconazole). The rate of ketoconazole binding is given by ΔA_430_−A_390_ (displacement of sterol (decrease in A_390_) due to ketoconazole binding (increase in A_430_)) and is an estimate for the rate at which the P450-sterol complex dissociated (*k*_off_).

### NaIO_4_ treatment of diols

The treatment of vicinal glycols with NaIO_4_ was performed *via* modification of a procedure reported by Ho *et al*. (62). P450 11A1 was incubated (0.5 s) with ^3^H-cholesterol according to the single-turnover (high Adx) conditions described above. The products of the reaction (extracted, dried, and dissolved in CH_3_OH (75 μl) were injected (45 μl) for analysis by radio-HPLC as described and the diol region (*t*_R_ 5.8 min to 7.3 min, Fig. 10) was collected into vials. The diols (in ∼1:9 H_2_O:CH_3_CN (v/v, ∼2 ml)) were extracted into CH_2_Cl_2_ (5 ml), the bottom layer (4 ml) was removed to a fresh vial, and the extraction process was repeated twice to maximize product recovery. The extracts (3 × 4 ml) were pooled and brought to dryness under a stream of nitrogen gas, and the dried residue was dissolved in CH_3_OH (100 μl). The diol sample was split (2 × 50 μl) between autosampler vials and one sample was treated with aqueous NaIO_4_ (2.5 mg ml^−1^) while the other was treated with H_2_O. Preliminary experiments with 20*R*,22*R*- and 20*R*,22*S*-(OH)_2_ cholesterol established that NaIO_4_ incubations were optimal with heating (37 °C) and that the reaction of the 20*R*,22*S*-(OH)_2_ cholesterol required substantially longer to go to completion (∼24 h) than did the natural substrate 20*R*,22*R*-(OH)_2_ (∼3 h). Accordingly, all NaIO_4_ incubations were performed at 37 °C for 24 h. After completion of the reaction, the samples were injected (20 μl) on radio-HPLC (Fig. S14) using the conditions described earlier.

### Kinetic modeling

Data were imported into KinTek Explorer software (v. 11.01, KinTek, https://kintekcorp.com/software) (63) as txt files and processed using an Apple computer (operating system 11.6.2). The indicated kinetic model (Table 2) was used with the enzyme and substrate concentrations, first estimating the rate of disappearance of the substrate cholesterol. Further adjustment of the individual rate constants was done to obtain the best fit, and the rate constants are reported (Figs. S15 and S16).

## Data availability

All data are available in the Supporting information in the form of synthetic procedures and characterization of chemicals, plus NMR, mass, and UV spectra. Also, key txt files include the data used for calculations for *k*_cat_, *K*_m_, and *K*_d_ determinations and fitting to kinetic models.

## Supporting information

This article contains supporting information (10, 21, 61).

## Conflict of interest

All of the authors declare that they have no conflict of interest with the contents of this article.
