# Supplementary material for: The multistep oxidation of cholesterol to pregnenolone by human cytochrome P450 11A1 is highly processive
Source: J Biol Chem. 2023 Nov 24;300(1):105495. doi: 10.1016/j.jbc.2023.105495 (PMC10716780; doi:10.1016/j.jbc.2023.105495)
Supplement: Supporting information [file mmc1.docx]

**Supporting Information**

The multi-step oxidation of cholesterol to pregnenolone by human cytochrome P450 11A1 is highly processive

Kevin D. McCarty, Lu Liu, Yasuhiro Tateishi, Hannah L. Wapshott-Stehli, and F. Peter Guengerich

Department of Biochemistry, Vanderbilt University School of Medicine, Nashville, Tennessee 37232-0146, United States

**Table of Contents**

**Synthetic procedures**

**Reagents**

**General**

**20,22-Dihydroxycholesterol**

**Step 1**

**Step 2**

**Step 3**

**Step 4**

**(20,22,22)-*d*_3_ Cholesterol**

**Step 1**

**Step 2**

**Step 3**

**Step 4**

**Step 5**

**Step 6**

**Step 7**

**Step 8**

**Step 9**

**Step 10**

**4-methylpentanal (isocaproaldehyde)**

**P450 11A1 nucleotide and amino acid sequences and vector map**

**Electrophoretic purity of enzymes, spectrum**

**Binding spectra of steroids- txt files for analysis**

**Kinetic analysis**

**txt files for analysis**

***k*_cat_, *K*_m_ determinations**

***K*_d_ determinations**

**Single turnover kinetics concentration files**

**Radio-HPLC trace**

**Screen shots for KinTek Explorer fitting**

**References**

**Synthetic procedures**

**Reagents.** Most chemicals were obtained from Sigma-Aldrich-Millipore, unless otherwise noted. D_2_ gas (stated 99.96% atomic excess, lecture bottle) was from Isotec/Millipore-Sigma-Aldrich.

**General.** NMR spectra were recorded on Bruker AV-400 or AV-II-600 MHz instruments in the Vanderbilt Small Molecular NMR Facility Core. Tetramethylsilane (TMS, δ 0.00 ppm) was used as an internal standard for ^1^H experiments, and an NMR solvent signal (δ 77.16 ppm for CDCl_3_) was used as a reference for ^13^C experiments. All mass spectra of sterols were collected (HRMS) using a Thermo Fisher Scientific LTQ XL Orbitrap mass spectrometer instrument in the APCI (positive ion) mode in the Vanderbilt Mass Spectrometry Research Core Facility, after introduction from UPLC using Waters ACQUITY ultra-performance liquid chromatography (UPLC) systems equipped with a ZORBAX Rx-C8 column (5 µm, 2.1 mm × 150 mm, Agilent) or an ACQUITY UPLC^®^ BEH C18 column (1.7 µm, 2.1 mm × 100 mm, Waters). Most spectra (sterols) were collected in the atmospheric pressure chemical ionization (APCI) mode. UV spectra were recorded either on-line from UPLC using a Waters Acquity instrument (diode array) or using an OLIS-Cary 14 spectrophotometer (On-Line Instrument Systems, Athens, GA) (in C_2_H_5_OH). Silicic acid chromatography was done using columns of SiliaFlash F60 (230-400 mesh). Analytical TLC was done with SilicaGel 60 F_254_ plates (from Merck KGaA), all with mixtures of hexanes and ethyl acetate, with visualization by UV light (254 nm) and spraying with an ethanolic phosphomolybdic acid solution and heating on a hot plate.

**Synthesis of 20,22-(OH)_2_ cholesterol (20*R*,22*R* and 20*R*,22*S*)**

The synthesis was based on that of Morisaki et al. (64) and a previous synthesis in this laboratory (10) (Fig. S1).

**Figure S1. Synthesis of 20,22-(OH)_2_ cholesterol.**

**Step 1.** **Pregnenolone 2-tetrahydropyranyl ether (2)** (65)

Pregnenolone (**1**, 6.32 g, 20 mmol) was dissolved in 96 ml of CH_2_Cl_2_ and stirred at 0 °C. Distilled 3,4-dihydropyran (9.1 ml, 8.4 g, 100 mmol) was added, along with 38 mg (0.18 mmol) of *p*-toluenesulfonic acid monohydrate. The reaction was stirred for 10 min at 0 °C and then 75 min at 23 °C. About one-half of the CH_2_Cl_2_ was removed *in vacuo* and replaced with (C_2_H_5_)_2_O, and the solution was sequentially washed with brine, saturated NaHCO_3_, and H_2_O (3× each). The solution was dried with anhydrous MgSO_4_, filtered, and concentrated *in vacuo* to give the product (7.8 g, 98% yield), which was used directly in the next step. LC-HRMS-APCI (*m/z*): [M+H–OTHP]^+^ calcd for C_21_H_31_O, 299.2369; found, 299.2350 (Δ –6.4 ppm).

**Step 2.** **(20*R*)-20-(1,3-Dithianyl-2)-pregn-5-ene-3β,20-diol 2-tetrahydropyranyl ether (3)**

Dithiane (5.1g, 43 mmol) was dissolved in 28 ml of tetrahydrofuran in a 250 ml round bottom flask and chilled to -20 °C (with a 3:1 (w/w) mixture of ice and NaCl). To this, with stirring, was added 18 ml of 2.5 M *n*-butyllithium in hexanes (45 mmol). The solution was brought to 23 °C and stirred for 1.5 h and then chilled to -20 °C again. The product **2** (from 7.8 g, Step 1) was dissolved in 28 ml of tetrahydrofuran. The solution was allowed to come to 23 °C and stirred overnight at 23 °C. Aqueous 2 M HCl (100 ml) was added and the product was extracted with 200 ml of ethyl acetate. The organic layer was washed (3×) with brine and then H_2_O, dried with Na_2_SO_4_, filtered, and concentrated *in vacuo*. The product (**3**) was crystallized from a mixture of hexanes and acetone—yield 5.9 g (58%). mp 193-196 °C (lit. 205-208 °C (64)), LC-HRMS-APCI (*m/z*): [M+H]^+^ calcd for C_30_H_47_O_2_S_2_, 503.3012; found, 503.3018 (Δ 0.1 ppm).

**Step 3.** **(20*R*)-20-Formylpregn-5-ene-3β,20-diol (4)**

Part of the product (**3**) (4.3 g, 8.3 mmol) from the preceding step was stirred with 11.5 g of HgCl_2_ (42 mmol) in 425 ml of a 4:1 (v/v) mixture of CH_3_CN and H_2_O, which was heated at reflux under Ar for 4 h. The mixture was cooled, and the liquid was decanted and diluted with ethyl acetate. The solution was washed 3× with 5 M NH_4_CH_3_CO_2_ and dried with anhydrous Na_2_SO_4_. The solvent was removed *in vacuo* and the product (**4**) was crystallized from acetone (2.1 g, 72% yield) mp 198-202 °C (literature 184-188 °C (64)). ^1^H NMR (600 MHz, CDCl_3_): δ 0.80 (s, 3H), 0.93-0.98 (m, 1H), 1.01 (s, 3H), 1.03-1.11 (m, 2H), 1.14-1.21 (m, 1H), 1.30 (dt, 1H, *J*=12.8, 4.0 Hz), 1.35 (s, 3H), 1.44 (d, 1H, *J*=4.2 Hz), 1.46-1.55 (m, 5H), 1.63-1.71 (m, 2H), 1.78 (t, 1H, *J*=9.8 Hz), 1.84-1.86 (m, 2H), 1.96-1.99 (m, 1H), 2.14 (d, 1H, *J*=12.6 Hz), 2.21-2.25 (m, 1H), 2.28-2.31 (m, 1H), 3.25 (s, 1H), 3.51-3.54 (m, 1H), 5.34-5.35 (m, 1H), 9.57 (s, 1H) (these were consistent with the previous report (64)). LC-HRMS-APCI (*m/z*): [M+H–H_2_O]^+^ calcd for C_22_H_33_O_2_, 329.2475; found, 329.2462 (Δ –4.0 ppm), [M+H–2H_2_O]^+^ calcd for C_22_H_31_O, 311.2369; found, 311.2355 (Δ –4.5 ppm).

**Step 4.** **Cholest-5-ene 3β,20,22-triol (5)**. A 25 ml solution of 2 M isoamyl magnesium bromide in (C_2_H_5_)_2_O (Sigma-Aldrich-Millipore) was diluted with 5 ml (C_2_H_5_)_2_O and 25 ml benzene and stirred at 0 °C. The aldehyde **4** (1.73 g, 5.0 mmol) was dissolved in a mixture of 40 ml tetrahydrofuran, 30 ml (C_2_H_5_)_2_O, and 30 ml benzene, and the above Grignard reagent was then added dropwise at 0 °C. The reaction was stirred at 0 °C, allowed to rise to 23 °C, and stirred for 15 h more. A solution of saturated aqueous NH_4_Cl was added, and the product was extracted into ethyl acetate. The solution was dried with Na_2_SO_4_, filtered, and concentrated *in vacuo.*

Crystallization of the residue from CH_3_OH yielded 244 mg of 20*R*,22*S*-(OH)_2_ cholesterol (**5b**). This product was then applied to a separate 2.0 cm × 30 cm silica column, which was washed with a 5:1 mixture of hexanes and ethyl acetate (v/v); the product was eluted with a 2:1 (v/v) mixture (207 mg, 10%). The residual material was purified on a 2.0 cm × 25 cm silica column, eluting sequentially with 10:1, 5:1, 2:1, and 1:1 mixtures of hexanes-ethyl acetate (all v/v, 350 ml each) to obtain 20*R*,22*R*-(OH)_2_ cholesterol (**5a**, 1.1 g, 55%). TLC (hexanes-ethyl acetate, 1:1, v-v) indicated that the 20*R*,22*R* product was eluted with the 2:1 hexanes-ethyl acetate mixture.

NMR spectra of the 20*R*,22*R* and 20*R*,22*S* products follow. A key difference is seen in the chemical shift of the H-22 doublet, which is at δ 3.39 in 20*R*,22*R*-(OH)_2_ cholesterol and at δ 3.23 in 20*R*,22*S*-(OH)_2_ cholesterol, in line with a literature precedent (66,67). The H-21 proton shifts are consistent with published work (21,64,66); i.e., Morisaki et al. (64) reported δ 1.20 for the 20*R*,20*S*-(OH)_2_, δ 1.26 for 20*R*,22*S*, and δ 1.05 for 20*S*,20*S*.

(20*R*,22*R*)-Cholest-5-ene-3β,20,22-triol (**5a**)

^1^H NMR (600 MHz, CDCl_3_): δ 0.89 (s, 3H), 0.90 (d, 6H, *J=*6.0 Hz), 0.92-0.96 (m, 1H), 0.99-1.02 (m, 1H), 1.02 (s, 3H), 1.05-1.10 (m, 1H), 1.12-1.18 (m, 1H), 1.19-1.22 (m, 2H), 1.22 (s, 3H), 1.24-1.28 (m, 1H), 1.41-1.57 (m, 11H), 1.61-1.66 (m, 1H), 1.80-.186 (m, 3H), 1.96-2.00 (m, 1H), 2.13 (dt, 1H, *J=*12.5, 3.4 Hz), 2.22-2.26 (m, 1H), 2.28-2.32 (m, 1H), 3.39 (d, 1H, *J=*9.0 Hz), 3.53 (m, 1H), 5.35-5.36 (m, 1H). ^13^C NMR (600 MHz, CDCl_3_): δ 13.7, 19.5, 20.5, 21.1, 22.1, 22.5, 23.1, 24.1, 28.2, 29.3, 31.4, 31.8, 31.9, 36.5, 36.6, 37.4, 40.3, 42.4, 43.3, 50.2, 54.9, 56.8, 71.9, 76.6, 77.6, 121.7, 140.9. LC-HRMS-APCI (*m/z*): [M+H–2H_2_O]^+^ calcd for C_27_H_43_O, 383.3308; found, 383.3304 (Δ –1.1 ppm).


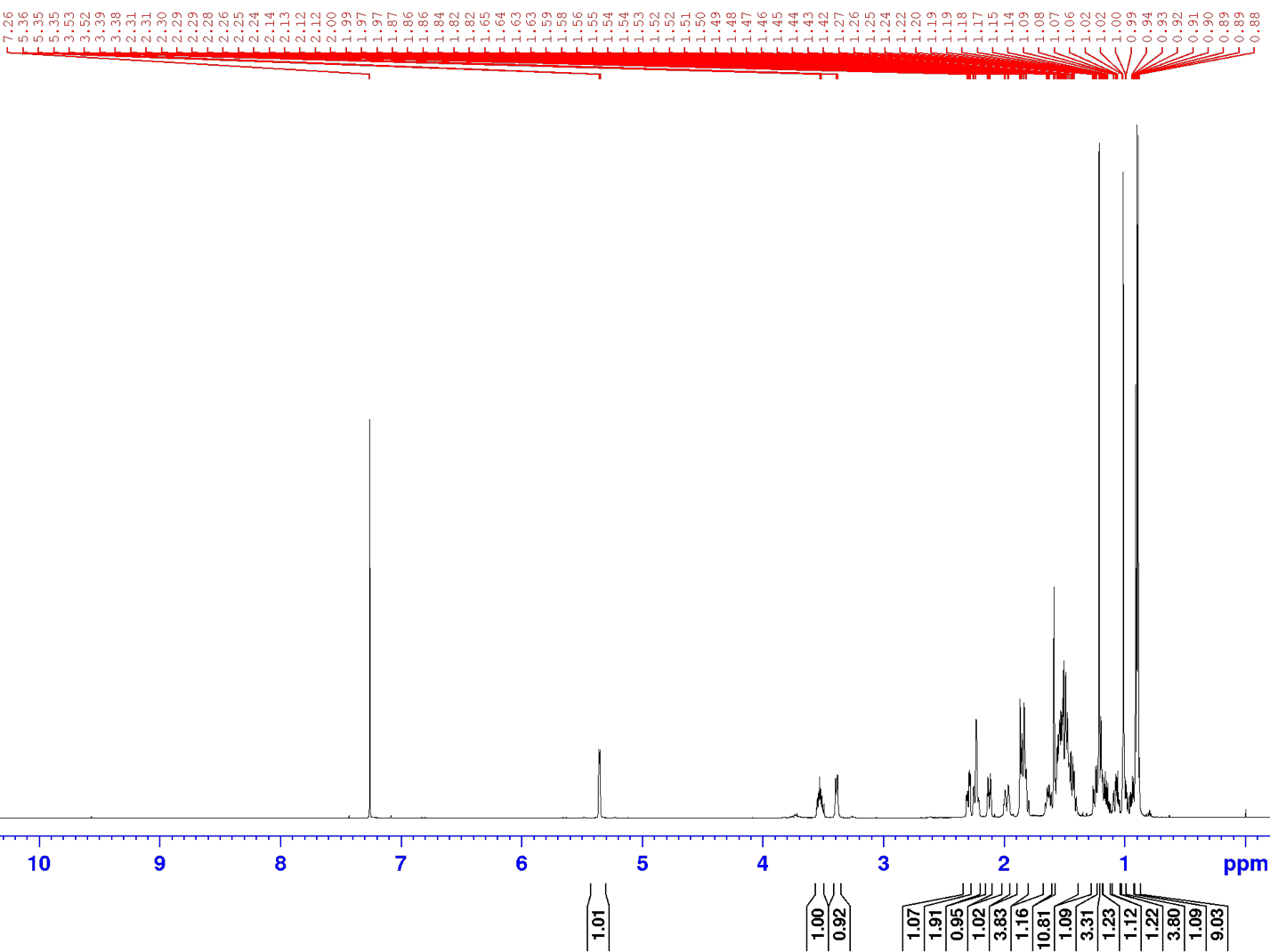


**Figure S2. ^1^H NMR spectrum of** **5a**


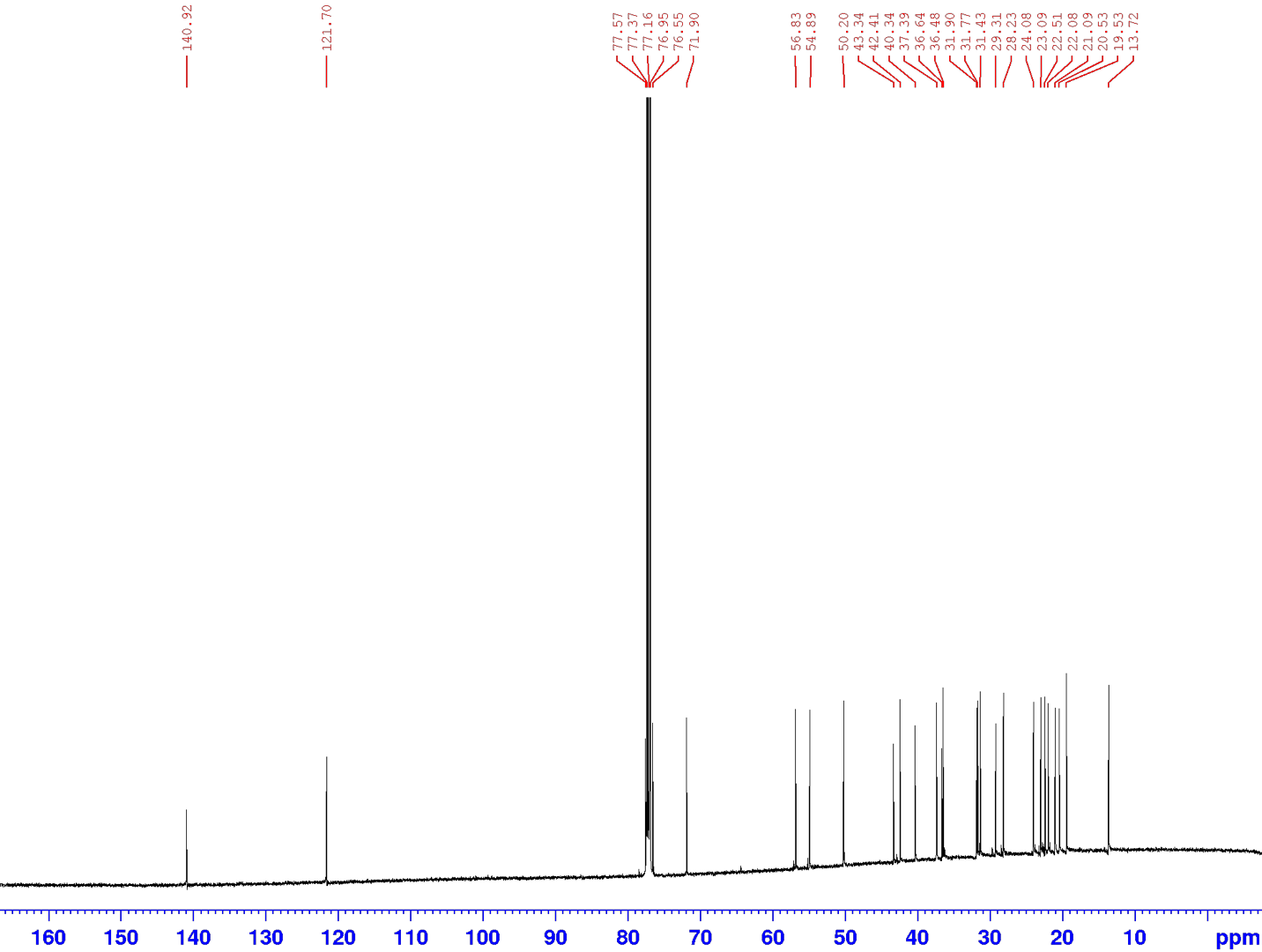


**Figure S3. ^13^C NMR spectrum of** **5a**

(20*R*,22*S*)-Cholest-5-ene 3β,20,22-triol (**5b**)

^1^H NMR (600 MHz, CDCl_3_): δ 0.88 (s, 3H), 0.90 (d, 3H, *J=*6.5 Hz), 0.91 (d, 3H, *J=*6.4 Hz), 0.92-0.96 (m, 1H), 0.98-1.04 (m, 1H), 1.01 (s, 3H), 1.05-1.10 (m, 1H), 1.11-1.16 (m, 1H), 1.18-1.21 (m, 1H), 1.22-1.25 (m, 2H), 1.27 (s, 3H), 1.42-1.60 (m, 8H), 1.61-1.73 (m, 6H), 1.77-1.80 (m, 1H), 1.83-1.86 (m, 2H), 1.96-2.00 (m, 1H), 2.10 (dt, 1H, *J=*12.6, 3.2 Hz), 2.22-2.26 (m, 1H), 2.29-2.31 (m, 1H), 3.25 (d, 1H, *J=*9.8 Hz), 3.50-3.55 (m, 1H), 5.35-5.36 (m, 1H). ^13^C NMR (600 MHz, CDCl_3_): δ 13.6, 19.5, 21.1, 22.6, 22.8, 22.9, 23.7, 24.3, 28.3, 29.5, 31.5, 31.8, 31.9, 36.60, 36.63, 37.4, 40.3, 42.4, 43.3, 50.2, 54.2, 56.5, 71.9, 76.6, 80.7, 121.8, 140.9. LC-HRMS-APCI (*m/z*): [M+H–2H_2_O]^+^ calcd for C_27_H_43_O, 383.3308; found, 383.3301 (Δ –2.0 ppm).


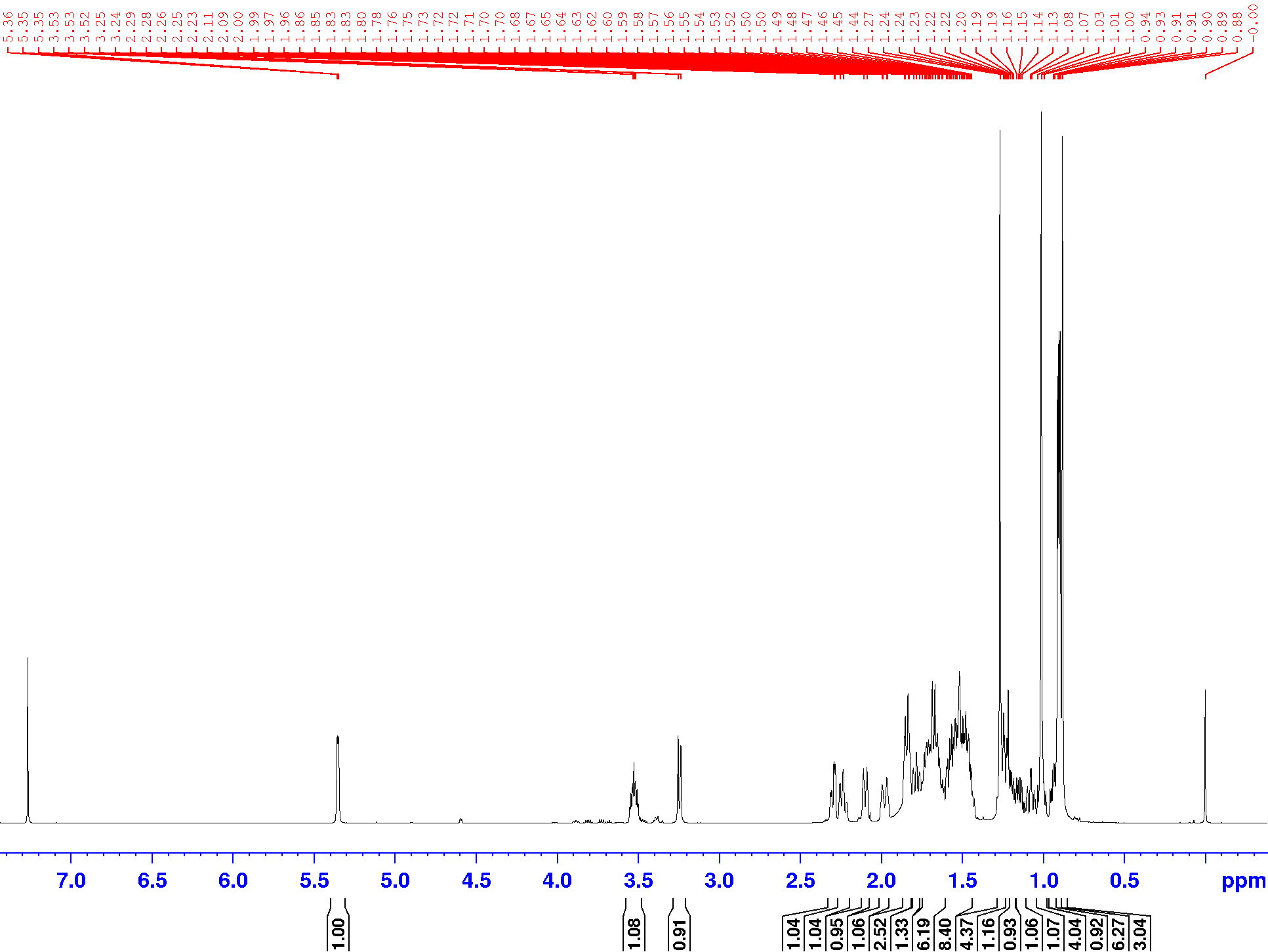


**Figure S4. ^1^H NMR spectrum of** **5b**


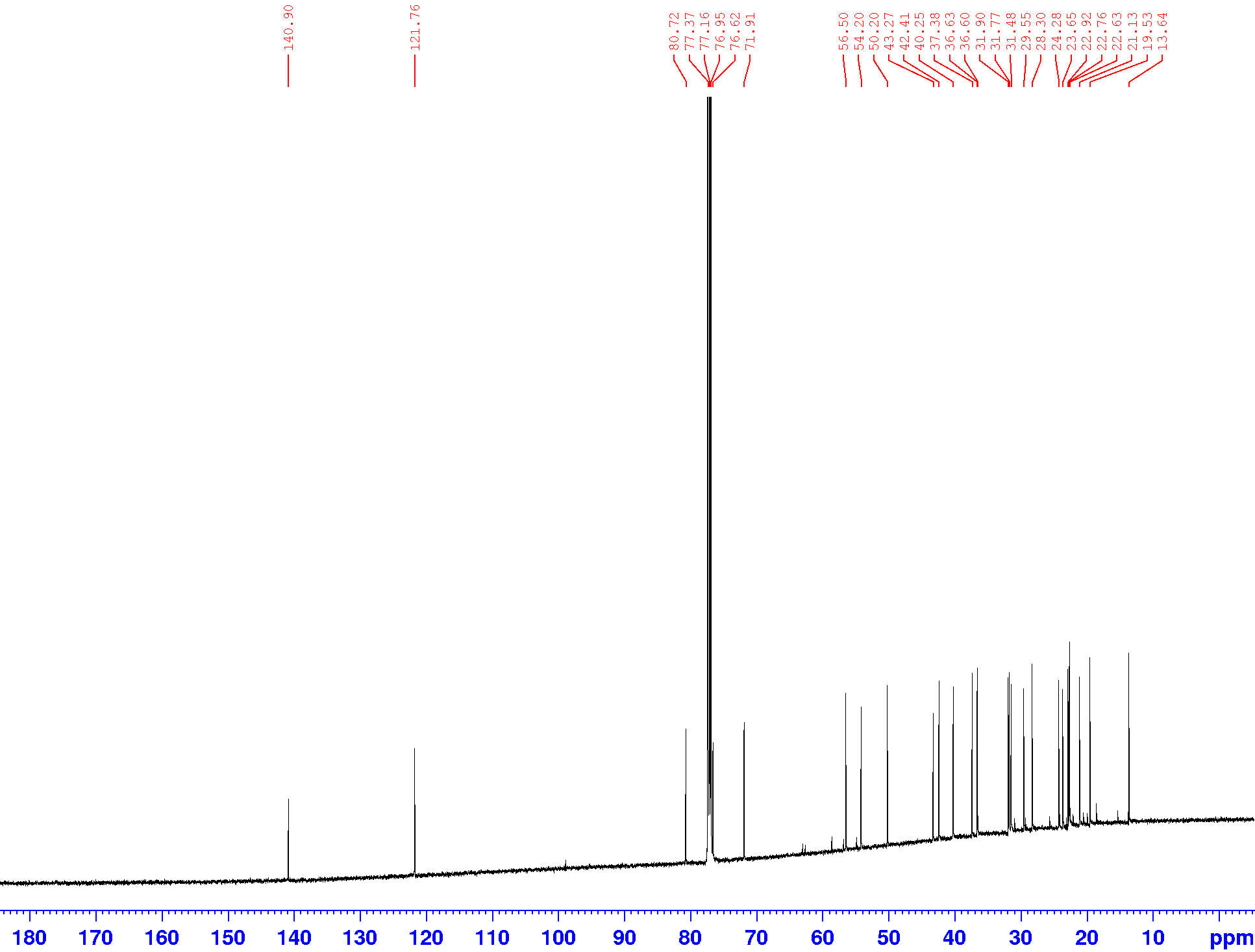


**Figure S5. ^13^C NMR spectrum of** **5b**

**Synthesis of 20,22-*d*_3_ cholesterol**

**Figure S6. Synthesis of (20,22,22)-*d*_3_ cholesterol (16).**

**Step 1. Ethyl 4-methylpentanoate (7)**

4-Methylpentanoic acid (**6**) (23.2 g, 0.20 mol) was stirred in 500 ml of C_2_H_5_OH and sparged with a stream of HCl gas. The mixture was heated at reflux overnight (with a drying tube filled with silica dessicant). Most of the C_2_H_5_OH was removed *in vacuo*, and the residue was dissolved in (C_2_H_5_)_2_O and washed sequentially with H_2_O, saturated NaHCO_3_, and H_2_O. The solution was dried with anhydrous MgSO_4_, filtered, and concentrated to give ethyl 4-methylpentanoate (25.0 g, 87% yield), which was used directly in the next step. ^1^H NMR (40 MHz, CDCl_3_): δ 0.88 (d, 6H, *J*=6.4 Hz), 1.23 (t, 3H, *J*=7.1 Hz), 1.48-1.59 (m, 3H), 2.27 (t, 2H, *J*=7.6 Hz), 4.10 (q, 2H, *J*=7.1 Hz).

**Step 2. 4-Methylpentan-1,1-*d*_2_-1-ol (8)**

 Ethyl 4-methylpentanoate (**7**, 13.8 g, 96 mmol, 15.0 ml) was stirred in 500 ml of (C_2_H_5_)_2_O (freshly opened can) in a 1-liter round-bottom flask, and solid LiAlD_4_ (Oakwood Chemicals, 2.0 g, 48 mmol) was added (23 °C) in aliquots over 60 min. The reaction was stirred overnight under reflux. After cooling, the reaction was quenched by the stepwise careful addition of 2 ml of H_2_O, 2 ml of 4 M NaOH, and 6 ml H_2_O (68). The mixture was filtered through paper, and the solution was washed 2× with 30 ml H_2_O. The (C_2_H_5_)_2_O layer with dried with anhydrous MgSO_4_, filtered, and concentrated carefully *in vacuo* and then under an N_2_ stream to 12 ml final volume (near theoretical yield). NMR analysis showed that the reaction was finished (no residual ester) and that deuteration of **7** at carbon 2 was complete ((C_2_H_5_)_2_O was still present and corrected for in calculating the yield (57%)). ^1^H NMR (600 MHz, CDCl_3_): δ 0.90 (d, 6H, *J*=6.7 Hz), 1.21-1.25 (m, 2 H), 1.56 (m, 3H). ^13^C NMR (150 MHz, CDCl_3_): δ 22.7, 28.0, 30.6, 35.0, 62.7 (quin, *J*=21 Hz).

**Step 3.** **4-Methylpentyl-1,1-*d*_2_ 4-methylbenzenesulfonate (9)** (69)

Compound **8** (5.54 g, 54 mmol) was dissolved in 170 ml of CH_2_Cl_2_ containing 38 ml (C_2_H_5_)_3_N (270 mmol) and 645 mg *N*,*N*-dimethyl-4-aminopyridine (5.4 mmol). Tosyl chloride (20.6 g, 108 mmol) was added, and the reaction was stirred overnight at 23 °C. The reaction was dissolved in ~ 500 ml of ethyl acetate, and the organic (upper) phase was washed 3× with brine. The product (**8**) was separated by chromatography on a 4.5 cm × 30 cm silica column, using sequential elution with 200 ml hexanes, 1800 ml 2% ethyl acetate in hexanes, and 1500 ml 5% ethyl acetate in hexanes (all v/v). Fractions were collected and monitored by TLC (silica, hexanes-ethyl acetate, 9-1, v/v, UV and phosphomolybdic acid-heat visualization). The residual tosyl chloride (*R*_f_ 0.54) and isoamyl tosylate (*R*_f_ 0.40) were separated (in the 2% ethyl acetate fractions) and the latter fractions were pooled and dried *in vacuo*. The ester (8.1 g, 31 mmol, 58% yield) had the correct spectral properties: UV (CH_3_OH) λ_max_ 224, 257, 263, 268, 274 nm; ^1^H NMR (400 MHz, CDCl_3_): δ 0.83 (d, 6H, *J*=6.6 Hz), 1.14-1.19 (m, 2H), 1.48 (septet, 1H, *J*=6.7 Hz), 1.62 (t, 2H, *J*=7.5 Hz), 2.45 (s, 3H), 7.34 (d, 2H, *J*= 8.0 Hz), 7.79 (d, 2H, *J*=8.3 Hz). HRMS-ESI (*m/z*): [M+H]^+^ calcd for C_13_H_19_D_2_O_3_S, 259.1331; found, 259.1330 (Δ –0.7 ppm).

**Step 4.** **4-Methylpentyl-1,1-*d*_2_ bromide (10)**

The tosylate product (**9**, 8.1 g, 31 mmol) was stirred with 4.1 g NaBr (39 mmol) in 50 ml of DMSO in a 100-ml round bottom flask at 23 °C for 90 h (70). The reaction mixture was extracted with three 30-ml portions of (C_2_H_5_)_2_O. The (C_2_H_5_)_2_O solution was washed with three 20-ml portions of ice-cold aqueous 10% H_2_SO_4_ (w/v), dried with anhydrous Na_2_SO_4_, and then carefully concentrated in vacuo and under an N_2_ stream (to 4.6 ml). ^1^H-NMR showed that the solution consisted of 4-methylpentyl bromide and residual (C_2_H_5_)_2_O in the molar ratio of 5:1 (yield 76%). ^1^H NMR (400 MHz, CDCl_3_): δ 0.90 (d, 6H, *J*=6.6 Hz), 1.28-1.34 (m, 2H), 1.53-1.61 (m, 1H), 1.85 (t, 2H, *J*=7.9 Hz).

**Step 5.** **(4-Methylpentyl-1,1-*d*_2_)triphenylphosphonium bromide (11)** (71,72).

4-Methylpentyl-1,1-*d*_2_ bromide (**10**, 4.1 g, 24 mmol, from step 4) was mixed with triphenylphosphine (Ph_3_P, 25 mmol, 6.55 g) in 25 ml of toluene, and the mixture was stirred and heated at reflux for 45 h. After cooling the mixture to room temperature, the triphenyl phosphonium bromide salt was collected on a Buchner funnel, under vacuum, and washed with benzene and then (C_2_H_5_)_2_O. The white solid was dried under vacuum for two days in a dessicator containing P_2_O_5_, under high vacuum, for the next step. Yield: 6.8 g, 70%.

**Step 6.** **(20*E*)-Cholesta-5,20(22)-dien-22-*d*-3β-ol (12) (*d*_1_) (Wittig Reaction)** (71,72)

All glass was dried at 200 °C in an oven and then cooled in a vacuum dessicator, and the reaction was done under an Ar atmosphere to avoid moisture. (4-Methylpentyl-1,1-*d*_2_)triphenylphosphonium bromide (**11**, 3.8 g, 9 mmol) was added to a 100-ml 3-necked round bottom flask, along with 9.7 ml of a 1.75 M solution of potassium *tert*-amylate in benzene (17 mmol), prepared from *tert*-amyl alcohol and potassium hydride exactly as described by Schow and McMorris (73) (and titrated with 1 M HCl, using pH indicator paper). The reaction mixture, which turned deep orange-red, was stirred and heated at reflux for 25 min (under Ar) to dissolve the reagents. Pregnenolone (790 mg, 2.5 mmol), dissolved in 20 ml of hot benzene, was added rapidly through one of the ports of the 3-neck flask. The mixture was stirred and heated under reflux for 2.5 h more, under an Ar balloon. The reaction was cooled and poured into 70 ml of H_2_O, which was extracted 3× with 50 ml of (C_2_H_5_)_2_O. The combined ether extracts were washed sequentially with H_2_O, cold 10% (v/v) H_2_SO_4_, and H_2_O, dried with anhydrous MgSO_4_, and concentrated in vacuo to give the product. The material was purified by chromatography on a 4 cm × 25 cm silica column, eluting sequentially with 4% (4500 ml), 8% (750 ml), and 15% (1000 ml) ethyl acetate in hexanes (v/v). The product eluted with 15% ethyl acetate, identified by TLC (silica, hexanes-ethyl acetate, 4-1, v/v; visualization with phosphomolybdic acid spray and heat). The fractions containing the product were combined and concentrated in vacuo to yield 869 mg of product (90% from pregnenolone).

Both the NMR and mass spectra suggested that this product was the mixture of 22-^1^H and 22-^2^H compounds. The 22-^2^H product was calculated to be 61%, from the peak area ratio of mass spectra. The product was determined to be the (*E*)-20,22-dehydrocholesterol by the shifts of the H-18 and H-21 proton singlets (δ 0.54 and 1.62, respectively, Fig. S7) (73). The C-18 singlet showed a minor component (~7%) at δ 0.66, indicative of the Z configuration (73). ^1^H NMR (600 MHZ, CDCl_3_): δ 0.54 (s, 3H), 0.88 (s, 3H), 0.89 (s, 3H), 1.01 (s,3H), 1.62 (s, 3H), 3.50-3.55 (m, 1H), 5.35-5.36 (m, 1H). LC-HRMS-APCI (*m/z*): [M+H–H_2_O]^+^ calcd for C_27_H_42_D, 368.3422; found, 368.3427 (Δ 1.5 ppm); calcd for C_27_H_43_, 367.3359; found, 367.3373 (Δ 3.8 ppm).


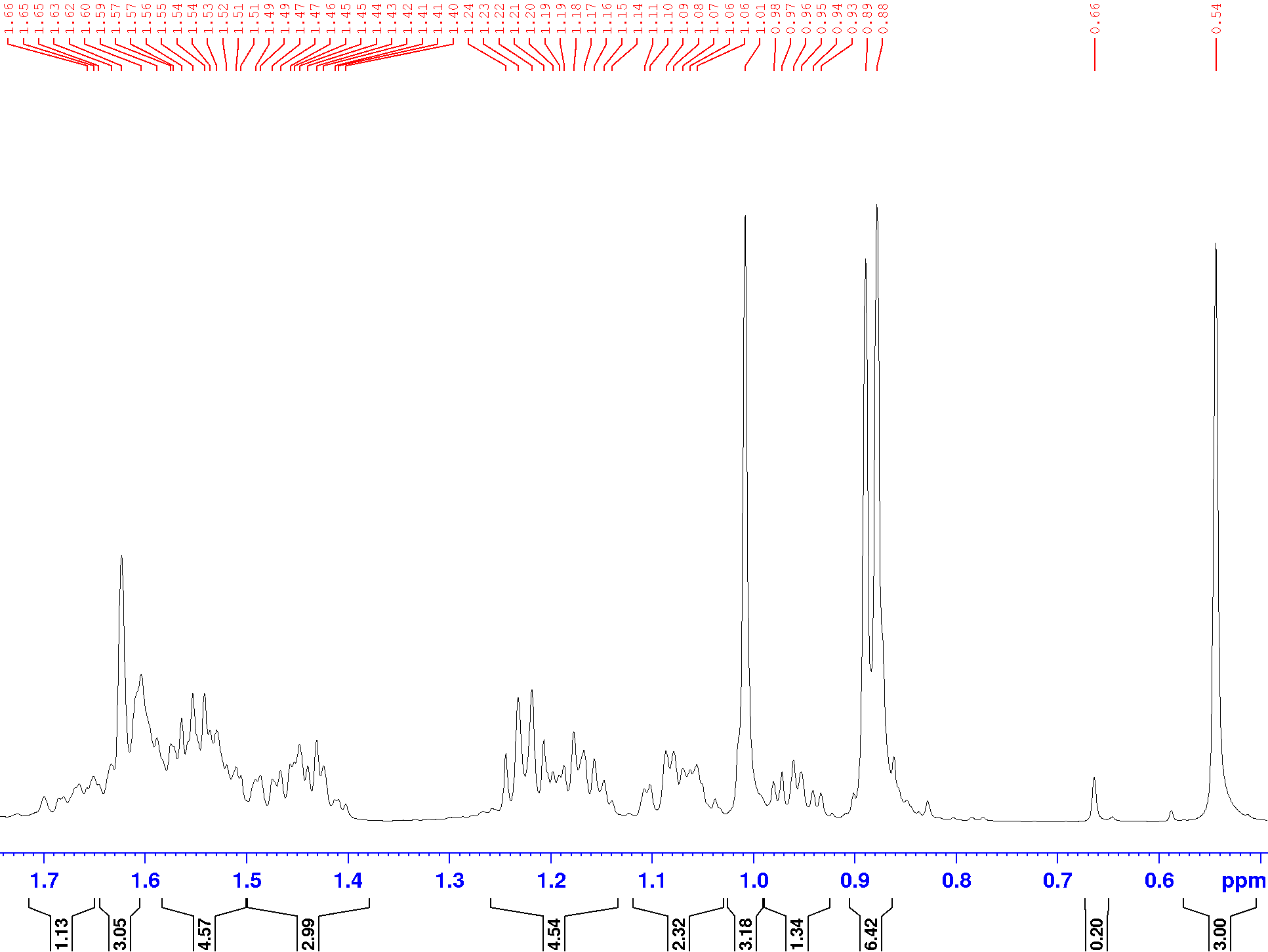


**Figure S7. ^1^H NMR spectra (expanded region between δ 0.5-1.70 ppm) of compound 12.**

**Step 7.** **(20*E*)-Cholesta-5,20(22)-dien-22-*d*-3β-ol acetate (13)**

(20*E*)-Cholesta-5,20(22)-dien-22-*d*-3β-ol (**12**, 102 mg) was mixed with 2 ml of pyridine and 2 ml of acetic anhydride and stirred overnight, in a round bottom flask equipped with a drying tube filled with silica gel. The mixture was poured into ice and allowed to thaw. (C_2_H_5_)_2_O (30 ml) was added and the phases were transferred to a separatory funnel. The (C_2_H_5_)_2_O layer was washed (3×) sequentially with H_2_O, cold 5% aqueous HCl (w/v), H_2_O, aqueous 5% NaHCO_3_ (w/v), and H_2_O. The (C_2_H_5_)_2_O phase was dried with MgSO_4_, filtered, and concentrated *in vacuo*. TLC (silica, hexanes-ethyl acetate, 4-1, v/v) showed complete acetylation (*R*_f_ 0.79, cf. 0.37 for starting material), and the product (**13**) was used directly in the next step. LC-HRMS-APCI (*m/z*): [M+H–AcOH]^+^ calcd for C_27_H_42_D, 368.3422; found, 368.3405 (Δ –4.8 ppm); calcd for C_27_H_43_, 367.3359; found, 367.3354 (Δ –1.3 ppm).

**Step 8.** **Cholest-5-ene-20,22,22-*d_3_*-3β-ol acetate (14) (*d*_3_)**

(20*E*)-Cholesta-5,20(22)-dien-22-*d*-3β-ol acetate (**13**, 102 mg, 0.24 mmol, from previous step) was dissolved in 6 ml of a 2% CH_3_CO_2_H in 1,4-dioxane solution (v/v) and stirred with 20 mg of PtO_2_ in a round bottom flack, fitted with a serum cap. D_2_ gas (5 ml, from a lecture bottle–Isotec, Millipore-Sigma-Aldrich, nominally 99.96% atom excess–fitted with a 2-stage regulator and transferred in a gas-tight syringe) was injected directly into the solution, and then a balloon of D_2_ (~ 500 ml) was attached to the flask with a glass inlet (72,73). After 4 h, 20 mg more PtO_2_ was added and the mixture was stirred under the D_2_ balloon (500 ml). The PtO_2_ was removed by filtration through a tuft of glass wool. The reaction was diluted with (C_2_H_5_)_2_O and the upper ((C_2_H_5_)_2_O) layer was washed sequentially with H_2_O, saturated NaHCO_3_, and H_2_O, dried with MgSO_4_, filtered, and concentrated in vacuo. LC-HRMS (C8 column, CH_3_CN) showed deuteration at the desired olefin and no further incorporation (i.e., 5,6-bond). LC-HRMS-APCI (*m/z*): [M+H–AcOH]^+^ calcd for C_27_H_42_D_3_, 372.3704; found, 372.3681 (Δ –6.3 ppm); C_27_H_43_D_2_, 371.3641; found, 371.3623 (Δ –5.1 ppm).

**Step 9.** **Cholest-5-ene-20,22,22-*d_3_*-3β-ol (15)**

The 3β-acetyl ester (**14**) from the previous step was dissolved in 5 ml of benzene and an equal volume of 2% (w/v) KOH in CH_3_OH was added. The solution was stirred and heated at 60 °C for 3 h under Ar. After cooling, 30 ml of (C_2_H_5_)_2_O was added and the upper phase was washed sequentially with 5% aq HCl, H_2_O, saturated NaHCO_3_, and H_2_O and then dried with MgSO_4_, filtered, and concentrated *in vacuo*. TLC (silica, hexanes-ethyl acetate, 4-1, v/v) showed that hydrolysis was complete. Yield 66 mg (71 % for steps 8 and 9 combined).

**Step 10.** **(20*R*)-Cholest-5-ene-20,22,22-*d_3_*-3β-ol (16) (*d*_3_)**.

The product with the same stereochemistry of natural cholesterol was purified from the epimeric cholesterol mixture (**15**) by preparative HPLC using a 10 mm × 250 mm octadecylsilane (C_18_) column (5 µm), eluting with CH_3_CN. LC-APCI-MS analysis indicated that this product is a mixture of *d*_3_, *d*_2_, *d*_1_, and *d*_0_ (cholesterol) products with 32%, 35%, 20%, and 13% abundance, respectively, as judged by their peak area ratios (Fig. S8). HRMS-APCI (*m/z*): [M+H–H_2_O]^+^ calcd for C_27_H_42_D_3_, 372.3704; found, 372.3710 (Δ +1.6 ppm).


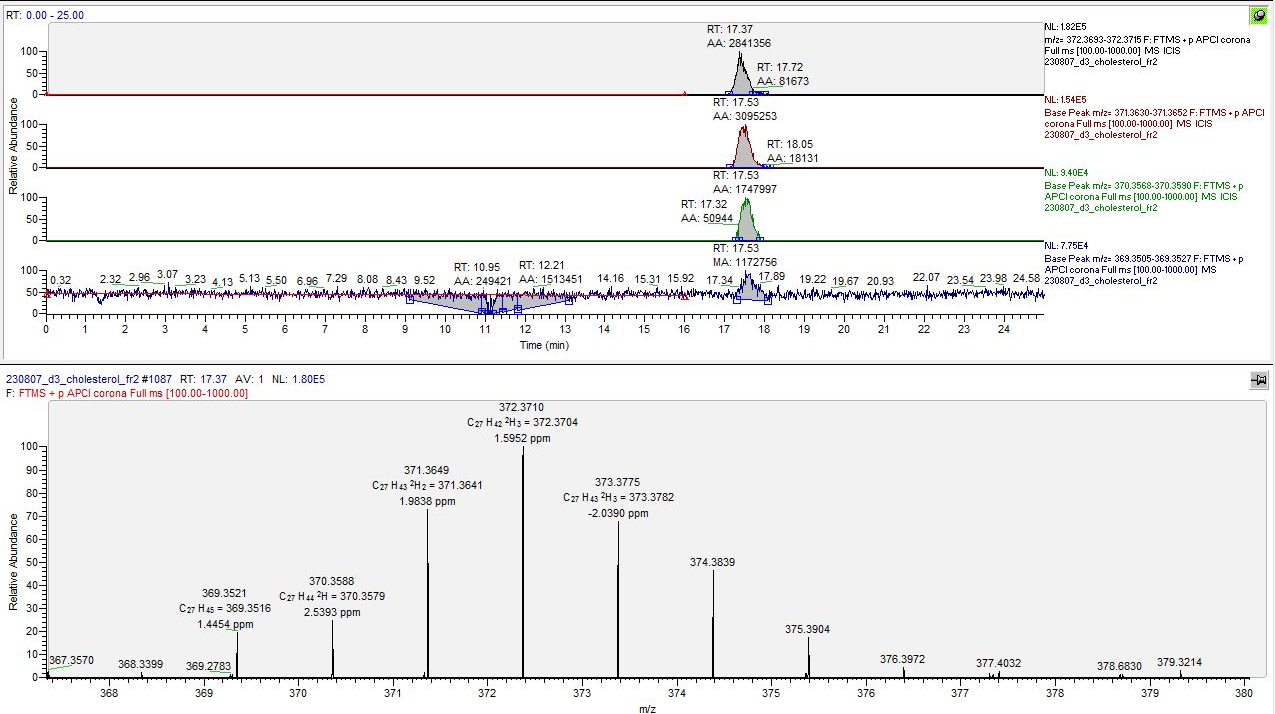


**Figure S8. LC-APCI-HRMS analysis of 16.**

The major issues regarding the lower deuterium content were (i) the lack of completely anhydrous conditions in the Wittig reaction (Step 6), as evidenced by the incorporation of protium into compound **12** (Fig. S6) and (ii) unanticipated incorporation of protium in the deuteration of compound **13** (step 8), even though the stated isotopic purity of the D_2_ gas was 99.96%. We attribute the low extent of labeling to the potential for scrambling known for heterogeneous hydrogenation catalysts (e.g., PtO_2_) (74,75), and possibly the trace acetic acid in the dioxane solvent. Despite these issues, the *d*_3_ component in the sample could be used under pre-state state (single turnover) conditions, in order to estimate the rate of cholesterol disappearance under first-order conditions.

**Synthesis of 4-methylpentanal (isocaproaldehyde)** (76)

A solution of 4-methylpentan-1-ol (613 mg, 6.0 mmol) and glacial CH_3_CO_2_ (50 µl) in CH_2_Cl_2_ (20 ml) was added dropwise to a solution of Dess-Martin periodinane (2.80 g, 6.6 mmol) in CH_2_Cl_2_ (20 ml), at room temperature for ~1 h. The resulting mixture was stirred for another 1 h before adding (C_2_H_5_)_2_O (140 ml), followed by the addition of 1.3 M NaOH (aq) (100 ml). After stirring at room temperature for 10 min, the organic layer was separated, washed with 1.3 M NaOH (aq) (50 ml), H_2_O (50 ml), and brine (50 ml), dried over anhydrous MgSO_4_, and evaporated to leave an oil. The crude material was purified with silicic acid column chromatography (CH_2_Cl_2_) to yield 4-methylpentanal **(**isocaproaldehyde) as a colorless oil (232 mg, 39% yield). ^1^H NMR (600 MHz, CDCl_3_): δ 0.90 (d, *J*=6.6 Hz, 6H), 1.52 (q, *J*=7.3 Hz, 2H), 1.54–1.62 (m, 1H), 2.43 (td, *J*=7.5, 1.4 Hz, 2H), 9.77 (t, *J*=1.7 Hz, 1H). ^13^C NMR (150 MHz, CDCl_3_): δ 22.4, 27.8, 30.9, 42.1, 203.1. These data are consistent with our previous report. (10)

**P450 11A1 nucleotide and amino acid sequences and vector map**

atg gct agt aca aga tca ccc agg cca ttt aat gag atc ccg agc ccg ggt gac aat

M   A   S   T   R   S   P   R   P   F   N   E   I   P   S   P   G   D   N

ggt tgg ctg aac ttg tac cat ttt tgg cgt gag act ggt acg cat aaa gtt cac ttg cac

G   W   L   N   L   Y   H   F   W   R   E   T   G   T   H   K   V   H   L   H

cac gtt cag aac ttc cag aaa tac ggc ccg atc tac cgc gag aaa ctg ggg aac gtg gaa

H   V   Q   N   F   Q   K   Y   G   P   I   Y   R   E   K   L   G   N   V   E

tct gtc tat gtt atc gat ccg gag gac gtc gcg ctg ctt ttt aaa agc gaa ggt ccg aat

S   V   Y   V   I   D   P   E   D   V   A   L   L   F   K   S   E   G   P   N

ccg gag cga ttc ctg att cca ccg tgg gtt gcg tat cac caa tat tac cag cgt cca atc

P   E   R   F   L   I   P   P   W   V   A   Y   H   Q   Y   Y   Q   R   P   I

ggc gtg ttg ctt aag aaa agc gca gcg tgg aag aag gac cgt gtg gcg ctc aat caa gag

G   V   L   L   K   K   S   A   A   W   K   K   D   R   V   A   L   N   Q   E

gtg atg gca ccg gag gcg acg aag aac ttt ctg ccg ctt tta gat gct gtg agc cgt gat

V   M   A   P   E   A   T   K   N   F   L   P   L   L   D   A   V   S   R   D

ttt gtg tcc gtt ctg cac cgt cgc atc aag aaa gct ggt tct ggc aac tac agc ggt gac

F   V   S   V   L   H   R   R   I   K   K   A   G   S   G   N   Y   S   G   D

atc agc gat gat ctg ttc aga ttc gcg ttt gag tcc att acg aac gtg atc ttt ggc gaa

I   S   D   D   L   F   R   F   A   F   E   S   I   T   N   V   I   F   G   E

cgt cag ggt atg ctg gag gaa gtg gtg aat ccg gag gcc cag cgc ttc att gac gcg att

R   Q   G   M   L   E   E   V   V   N   P   E   A   Q   R   F   I   D   A   I

tat cag atg ttt cac acc tcg gta ccg atg ttg aac ctg ccg ccg gac ctc ttc cgc ctc

Y   Q   M   F   H   T   S   V   P   M   L   N   L   P   P   D   L   F   R   L

ttc cgc acc aaa acc tgg aag gat cat gtt gcg gcg tgg gat gta atc ttc agc aag gcc

F   R   T   K   T   W   K   D   H   V   A   A   W   D   V   I   F   S   K   A

gat atc tac acc caa aac ttt tat tgg gaa ctg cgt cag aaa ggt agc gtt cat cac gac

D   I   Y   T   Q   N   F   Y   W   E   L   R   Q   K   G   S   V   H   H   D

tac cgc ggt att ctg tat cgt ctg ctg ggc gac agc aag atg agc ttc gag gac atc aag

Y   R   G   I   L   Y   R   L   L   G   D   S   K   M   S   F   E   D   I   K

gct aat gtg acc gaa atg ctg gcg ggc ggt gtt gat acc acg tcc atg acc ctg caa tgg

A   N   V   T   E   M   L   A   G   G   V   D   T   T   S   M   T   L   Q   W

cat ctg tac gag atg gct cgt aat tta aag gtc caa gat atg ctg cgt gca gaa gtg ttg

H   L   Y   E   M   A   R   N   L   K   V   Q   D   M   L   R   A   E   V   L

gcg gct cgt cat cag gca cag ggc gac atg gcg acc atg ttg cag ctg gtt ccg ctg ttg

A   A   R   H   Q   A   Q   G   D   M   A   T   M   L   Q   L   V   P   L   L

aag gcg agc att aaa gaa acc ctg cgt tta cac ccg atc tct gtc acc ctg caa cgc tat

K   A   S   I   K   E   T   L   R   L   H   P   I   S   V   T   L   Q   R   Y

ctg gtt aat gac ttg gtg tta cgt gat tac atg att ccg gca aaa acc ttg gtg caa gtt

L   V   N   D   L   V   L   R   D   Y   M   I   P   A   K   T   L   V   Q   V

gcg att tac gcc ctg ggc cgt gaa ccg acc ttc ttt ttc gac ccg gag aac ttt gat cca

A   I   Y   A   L   G   R   E   P   T   F   F   F   D   P   E   N   F   D   P

acg aga tgg ctg agc aag gac aaa aat att acc tat ttc cgc aac ctg ggc ttt ggt tgg

T   R   W   L   S   K   D   K   N   I   T   Y   F   R   N   L   G   F   G   W

ggt gtt cgt caa tgc ctg ggc cgt cgt atc gcc gaa ctg gag atg acc att ttc ctg atc

G   V   R   Q   C   L   G   R   R   I   A   E   L   E   M   T   I   F   L   I

aac atg ctg gaa aac ttc cgc gtt gaa atc cag cat ctg tcc gac gtt ggt aca act ttc

N   M   L   E   N   F   R   V   E   I   Q   H   L   S   D   V   G   T   T   F

aac ttg att ctg atg ccg gaa aaa ccg atc tcg ttc acc ttt tgg cct ttt aac caa gaa

N   L   I   L   M   P   E   K   P   I   S   F   T   F   W   P   F   N   Q   E

gcg acg cag cag cac cat cac cac cac cat ta

A   T   Q   Q   H   H   H   H   H   H

**Figure S9. Nucleotide and amino acid sequences of the histidine-tagged P450 11A1 construct.** Nucleic acid sequence was codon-optimized for expression in *Escherichia coli*.


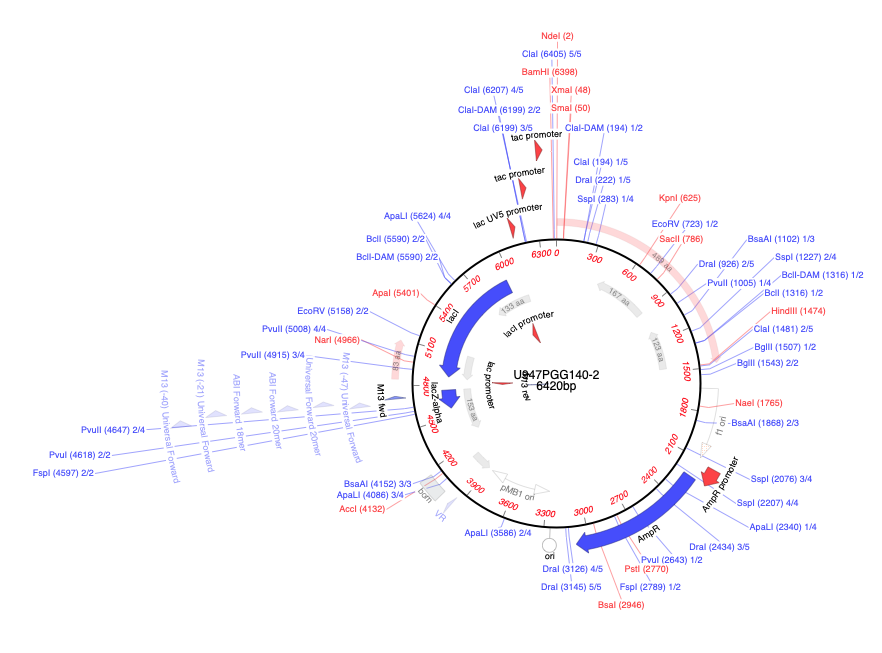


**Figure S10. Vector map of P450 11A1 in pET23C+. The nucleic acid sequence corresponding to the 489 amino acid sequence of P450 11A1 is indicated between NdeI and HindIII with an orange arrow (labeled “489 aa”).**

**Figure S11. Electrophoretic purity of P450 11A1.** SDS polyacrylamide gel electrophoresis was done using a 4-12% gradient gel (staining with Coomassie Brilliant Blue). The lanes are labeled, and the masses of the Mr standards are listed on the right edge of the photograph.

**Figure S12. Reduced CO spectrum of purified P450 11A1.** Fe^2+^-CO vs. Fe^2+^ (61,77). The concentration was 6.0 µM.

**Kinetic analysis**

**txt files for analysis**

***k*_cat_, *K*_m_ determinations**

From Fig. 5 for analysis.

Substrate: Cholesterol

[Chol](µM) Rates, min-1

7.5 1.32 1.18

15.0 1.88 1.72

20.0 2.38 2.20

30.0 2.47 2.46

40.0 3.96 3.88

50.0 4.06 4.00

75.0 4.99 4.93

100.0 5.93 4.92

150.0 4.83 5.08

Substrate: 22*R*-OH Cholesterol

[22R](µM) Rates, min-1

2.50 1.34 1.02

5.00 1.68 1.48

7.50 1.94 2.15

15.00 3.15 3.07

20.00 3.29 3.38

30.00 4.66 5.17

40.00 5.08 5.08

50.00 5.23 5.80

75.00 6.76 6.27

100.00 7.41 6.71

150.00 7.68 8.05

Substrate: 20*R*,22*R*-(OH)_2_ Cholesterol

[20R,22R](µM) Rates, min-1

5.0 2.46 2.24

7.5 2.57 2.85

15.0 3.83 4.18

20.0 4.26 5.83

30.0 5.47 6.23

40.0 7.20 6.21

50.0 6.51 8.53

75.0 7.45 10.26

100.0 12.01 10.27

150.0 9.70 11.61

Substrate: 20*R*,22*S*-(OH)_2_ Cholesterol

[20R,22S](µM) Rates, min-1

7.50 2.07 1.68

15.00 1.97 1.99

20.00 2.93 2.88

30.00 3.59 3.11

40.00 3.90 4.10

50.00 3.70 3.57

75.00 4.16 4.38

100.00 4.14 4.09

150.00 4.64 4.37

***K*_d_ determinations.**

From Fig. 6. Txt files for analysis

Ligand: Cholesterol (-Adx)

[Chol](µM) A387.2-A419.6

0.000 0.001

0.050 0.027

0.100 0.045

0.150 0.059

0.200 0.068

0.250 0.073

0.375 0.082

0.500 0.087

0.750 0.098

1.000 0.103

1.250 0.098

1.500 0.105

2.000 0.109

2.500 0.109

3.750 0.117

5.000 0.115

10.000 0.117

Ligand: Cholesterol (+Adx)

[Chol](µM) A387.2 - A418.8

0.000 -6.913e-005

0.050 0.028

0.100 0.046

0.150 0.062

0.200 0.068

0.250 0.076

0.375 0.087

0.500 0.090

0.750 0.101

1.000 0.106

1.250 0.105

1.500 0.109

2.000 0.115

2.500 0.117

3.750 0.117

5.000 0.114

10.000 0.121

Ligand: 22*R*-OH Cholesterol (-Adx)

[22R](µM) A394.8-424.8

0. 0.002

0.050 0.024

0.100 0.042

0.150 0.047

0.200 0.050

0.250 0.053

0.375 0.054

0.500 0.053

0.750 0.055

1.000 0.054

1.250 0.056

1.500 0.058

2.000 0.056

3.250 0.065

4.500 0.060

9.500 0.058

Ligand: 22*R*-OH Cholesterol (+Adx)

[22R](µM) A394.8-A424.8

0.000 0.002

0.050 0.024

0.100 0.042

0.150 0.047

0.200 0.050

0.250 0.053

0.375 0.054

0.500 0.053

0.750 0.055

1.000 0.054

1.250 0.056

1.500 0.058

2.000 0.056

3.250 0.065

4.500 0.060

9.500 0.058

Ligand: 20*R,*22*R*-(OH)_2_ Cholesterol (-Adx)

[20R,22R](µM) A389.2- A420

0.000 0.003

0.050 0.064

0.100 0.071

0.150 0.072

0.200 0.077

0.250 0.078

0.375 0.078

0.500 0.080

0.750 0.085

1.000 0.089

1.250 0.090

1.500 0.091

2.000 0.095

2.500 0.099

3.750 0.106

5.000 0.107

10.000 0.107

Ligand: 20*R,*22*R*-(OH)_2_ Cholesterol

[20R,22R](µM) A388-A418.8

0.000 0.001

0.050 0.054

0.100 0.071

0.150 0.078

0.200 0.084

0.250 0.087

0.375 0.090

0.500 0.091

0.750 0.101

1.000 0.103

1.250 0.106

1.500 0.109

2.000 0.110

2.500 0.111

3.750 0.114

5.000 0.115

10.000 0.117

Ligand: 20*R,*22*S*-(OH)_2_ Cholesterol (-Adx)

[20R,22S](µM) A389.6-A422.4

0.000 0.002

0.050 0.018

0.100 0.022

0.150 0.025

0.200 0.027

0.250 0.029

0.375 0.031

0.500 0.032

0.745 0.035

0.990 0.038

1.235 0.040

1.480 0.040

1.970 0.041

2.460 0.041

3.685 0.044

4.910 0.044

9.810 0.045

Ligand: 20*R,*22*S*-(OH)_2_ Cholesterol (+Adx)

[20R,22S](µM) A388 - A422.8

0.000 1.535e-004

0.050 0.013

0.100 0.019

0.150 0.024

0.200 0.027

0.250 0.027

0.375 0.030

0.500 0.031

0.745 0.034

0.990 0.037

1.235 0.037

1.480 0.038

1.970 0.037

2.460 0.041

3.685 0.041

4.910 0.043

9.810 0.044

Ligand: Pregnenolone (-Adx)

[Preg]µM A385.2 - A416.8

0.000 0.001

0.050 0.003

0.100 0.005

0.150 0.005

0.200 0.008

0.250 0.005

0.375 0.010

0.500 0.011

0.750 0.012

1.000 0.012

1.250 0.011

1.500 0.015

2.000 0.014

2.500 0.020

3.750 0.016

5.000 0.022

10.000 0.016

Ligand: Pregnenolone (+Adx)

[Preg] (µM) A372 - A415.2

0.000 0.002

0.050 0.005

0.100 0.004

0.150 0.006

0.200 0.008

0.250 0.009

0.375 0.010

0.500 0.012

0.750 0.015

1.000 0.014

1.250 0.017

1.500 0.017

2.000 0.021

2.500 0.020

3.750 0.019

5.000 0.020

10.000 0.025

**Single turnover kinetics concentration files**

Reaction: P450 11A1 + 10-fold excess (high) Adx

| Time, s | 20R,22R-(OH)_2_ (µM) | 22R-OH (µM) | PREG  (µM) | CHOL  (µM) |
| --- | --- | --- | --- | --- |
| 0 | 0 | 0 | 0 | 4.5 |
| 0.25 | 0 | 0.166491 | 0 | 4.333509 |
| 0.5 | 0.136594 | 0.341085 | 0 | 4.022322 |
| 0.75 | 0.240693 | 0.257658 | 0.136781 | 3.864868 |
| 1 | 0.227848 | 0.20981 | 0.314241 | 3.748101 |
| 2 | 0.167248 | 0.214374 | 0.68655 | 3.431828 |
| 2.5 | 0.205084 | 0.164892 | 0.764686 | 3.365338 |
| 3.5 | 0.183999 | 0.165224 | 1.037342 | 3.113435 |
| 5 | 0.191132 | 0.157568 | 1.310888 | 2.840412 |
| 6 | 0.186544 | 0.166593 | 1.384615 | 2.762248 |
| 7.5 | 0.209505 | 0.164767 | 1.61821 | 2.507517 |
| 10 | 0.181663 | 0.159068 | 2.34535 | 1.813918 |
| 15 | 0.152214 | 0.135052 | 2.972641 | 1.240093 |
| 20 | 0.110612 | 0.100994 | 3.346265 | 0.942129 |
| 30 | 0.034217 | 0.097387 | 3.763014 | 0.605381 |
| 45 | 0 | 0.069281 | 4.002393 | 0.428326 |
| 60 | 0 | 0.06822 | 4.115624 | 0.316156 |
| 90 | 0 | 0.050627 | 4.18942 | 0.259952 |

Reaction: P450 11A1 + 10-fold excess (high) Adx

| Time, s | 20R,22R-(OH)_2_ (µM) | 22R-OH (µM) | PREG (µM) | CHOL (µM) |
| --- | --- | --- | --- | --- |
| 0 | 0 | 0 | 0 | 4.5 |
| 10 | 0.23903 | 0.226239 | 1.240718 | 2.794013 |
| 25 | 0.161929 | 0.176335 | 2.512486 | 1.649251 |
| 50 | 0.071945 | 0.117221 | 3.582386 | 0.728447 |
| 75 | 0.047869 | 0.062549 | 4.057372 | 0.33221 |
| 100 | 0 | 0 | 4.340924 | 0.159076 |
| 200 | 0 | 0 | 4.5 | 0 |
| 500 | 0 | 0 | 4.5 | 0 |

**Figure S13. Steady-state kinetic assay of P450 11A1 with [1,2-^3^H]-cholesterol.** The enzyme reaction was performed in triplicate as described for the steady-state LC-MS assays, but with the use of ^3^H-cholesterol (2 mCi mmol^-1^) as the substrate. Product (^3^H-pregnenolone) was detected by radio-HPLC as described (Experimental Methods). The highest rate measured was 6.7 ± 0.4 nmol product min^-1^ (nmol P450)^-1^.

**Figure S14. Isolation and NaIO_4_ treatment of cholesterol diols.** *A*, the products of a 0.5 s reaction of P450 11A1 with ^3^H-cholesterol. *B*, The diol region from Part *A* (*t*_R_ 5.8 min to 7.5 min) was isolated by HPLC and half of the sample was treated with NaIO_4_ (see Methods). After 24 h of incubation, the samples were re-injected on radio-HPLC, by which point all of the slower eluting diol peak (*t*_R_ 6.8 min) had converted to the faster eluting peak (*t*_R_ 6.2 min, see -NaIO_4_ treatment (in black)). Neither peak remained after treatment with NaIO_4_ (in red)-pregnenolone eluted at *t*_R_ 3.5 min.

**KinTek Explorer fitting**

**
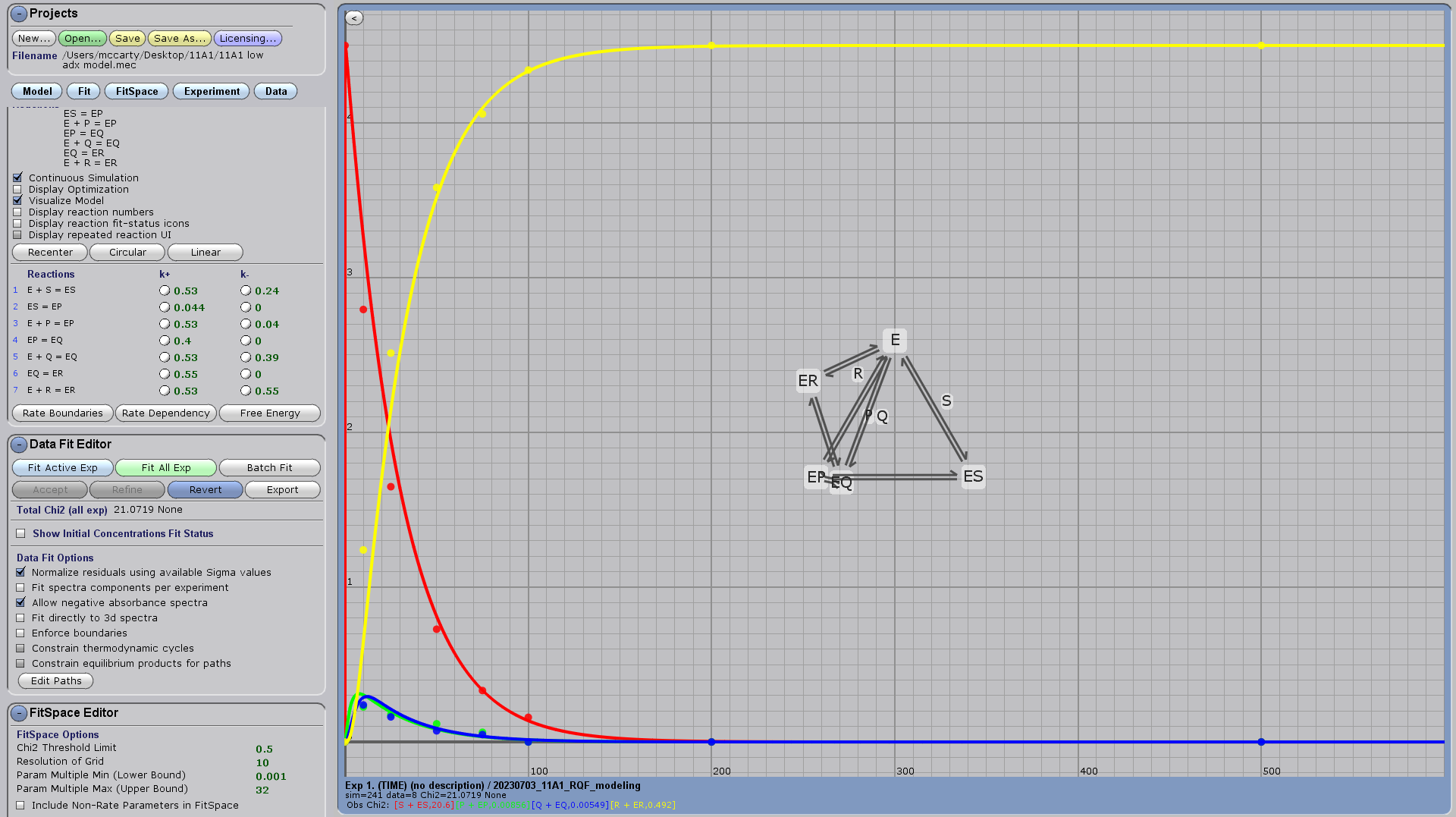
**

**Fig S15. Kinetic modeling of a P450 11A1 single-turnover reaction (low Adx).** Raw data constituting Fig. 9*A*. Kinetic parameters and model input are in the column (left side).

**
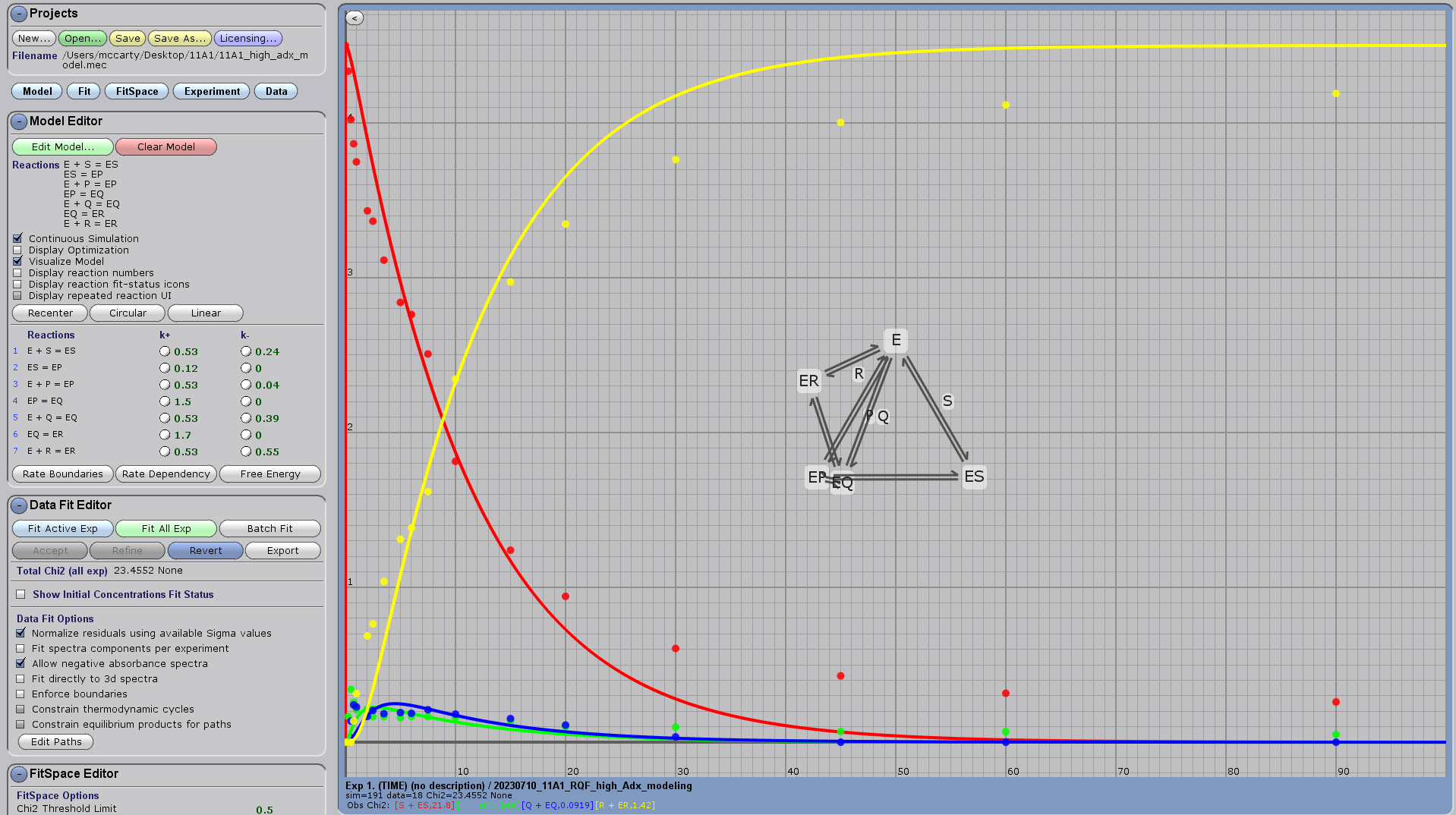
**

**Figure S16. Kinetic modeling of a P450 11A1 single-turnover reaction (high Adx).** Raw data constituting Fig. 9*B*. Kinetic parameters and model input are in the column (left side).

References for Supporting Information section

10. Yoshimoto, F. K., Jung, I. J., Goyal, S., Gonzalez, E., and Guengerich, F. P. (2016) Isotope-labeling studies support the electrophilic Compound I iron active species, FeO^3+^, for the carbon-carbon bond cleavage reaction of the cholesterol side-chain cleavage enzyme, cytochrome P450 11A1. *J. Am. Chem. Soc.* **138**, 12124-12141

21. Sugano, S., Miura, R., and Morishima, N. (1996) Identification of intermediates in the conversion of cholesterol to pregnenolone with a reconstituted cytochrome P-450_scc_ system: accumulation of the intermediate modulated by the adrenodoxin level. *J. Biochem. (Tokyo)* **120**, 780-787

61. Guengerich, F. P. (2014) Analysis and characterization of enzymes and nucleic acids relevant to toxicology. in *Hayes' Principles and Methods of Toxicology* (Hayes, A. W., and Kruger, C. L. eds.), 6th Ed., CRC Press-Taylor & Francis Boca Raton, FL. pp 1905-1964

64. Morisaki, M., Sato, S., and Ikekawa, N. (1977) Studies on steroids. XLV. Synthesis of the four stereoisomers of 20, 22-dihydroxycholesterol. *Chem. Pharm. Bull.* **25**, 2576-2583

65. Bernady, K. F., Floyd, M. B., Poletto, J. F., and Weiss, M. J. (1979) Prostaglandins and congeners. 20. Synthesis of prostaglandins via conjugate addition of lithium *trans*-1-alkenyltrialkylalanate reagents. A novel reagent for conjugate 1,4-additions. *J. Org. Chem.* **44**, 1438-1447

66. Ruan, B., Wilson, W. K., and Schroepfer, G. J., Jr. (1999) An improved synthesis of (20*R*,22*R*)-cholest-5-ene-3β,20,22-triol, an intermediate in steroid hormone formation and an activator of nuclear orphan receptor LXRα. *Steroids* **64**, 385-395

67. Chaudhuri, N. K., Nickolson, R., Kimball, H., and Gut, M. (1970) The synthesis and stereochemistry of (22*R*)-20α,22- and (22*S*)-20α, 22-dihydroxycholesterol. *Steroids* **15**, 525-539

68. Fieser, L. F., and Fieser, M. (1967) *Reagents for Organic Synthesis, Vol. 1*, Wiley, New York, pp 581-595

69. Baek, D. J., and Bittman, R. (2013) Synthesis of cholesterol analogs having varying length alkyl side chains including cholesterol-23, 23, 24, 24, 25, 26, 26, 26, 27, 27, 27-d(11) as probes of cholesterol's functions and properties. *Chem. Phys. Lipids* **175-176**, 99-104

70. Cason, J., and Correia, J. S. (1961) Investigation of methods for preparing pure secondary alkyl halides. *J. Org. Chem.* **26**, 3645-3649

71. Schmit, J. P., Piraux, M., and Pilette, J. F. (1975) Application of the Wittig reaction to the synthesis of steroidal side chains. Possibility of 3β-phenoxy formation as a secondary reaction. *J. Org. Chem.* **40**, 1586-1588

72. Caballero, G. M., and Gros, E. G. (1994) Synthesis of [21-^13^C]-cholesterol. *J. Label. Compd. Radiopharm.* **34**, 127-130

73. Schow, S. R., and McMorris, T. C. (1979) Utility of the Wittig reaction for the construction of side chains of steroids starting from pregnenolone. *J. Org. Chem.* **44**, 3760-3765

74. Carey, F. A., and Sundberg, R. J. (1990) *Advanced Organic Chemistry*, 3rd ed., Part B, Plenum Press, New York, pp 219-230

75. Smith, M. B., and March, J. (2007) *March's Advanced Organic Chemistry: Reactions, Mechanisms, and Structure*, 6th ed., Wiley-Interscience, New York, pp 1053-1062

76. Kouji, H., and Yoshimori, A. (2022) Novel peptidomimetic compound and computer design. WO2022124341 (patent)

77. Omura, T., and Sato, R. (1964) The carbon monoxide-binding pigment of liver microsomes. I. Evidence for its hemoprotein nature. *J. Biol. Chem.* **239**, 2370-2378
